# NIR-II-driven and glutathione depletion-enhanced hypoxia-irrelevant free radical nanogenerator for combined cancer therapy

**DOI:** 10.1186/s12951-021-01003-2

**Published:** 2021-09-06

**Authors:** Li Zhang, Yadi Fan, Zhe Yang, Mo Yang, Chun-Yuen Wong

**Affiliations:** 1 Department of Chemistry, City University of Hong Kong, Tat Chee Avenue, Kowloon, Hong Kong SAR; 2 Department of Biomedical Engineering, The Hong Kong Polytechnic University, Hung Hom, Kowloon, Hong Kong SAR; 3 State Key Laboratory of Terahertz and Millimeter Waves, City University of Hong Kong, Tat Chee Avenue, Kowloon, Hong Kong SAR

**Keywords:** Hypoxia-irrelevant, Glutathione-depleting, Hydroxyl radical, NIR-II photothermal therapy, Alkyl radical

## Abstract

**Background:**

Though the combination of photodynamic therapy (PDT) and chemodynamic therapy (CDT) appears to be very attractive in cancer treatment, hypoxia and overproduced glutathione (GSH) in the tumor microenvironment (TME) limit their efficacy for further application.

**Results:**

In this work, a smart hypoxia-irrelevant free radical nanogenerator (AIPH/PDA@CuS/ZIF-8, denoted as APCZ) was synthesized in situ via coating copper sulphide (CuS)-embedded zeolitic imidazolate framework-8 (ZIF-8) on the free radical initiator 2,2′-azobis[2-(2-imidazolin-2-yl)propane]-dihydrochloride (AIPH)-loaded polydopamine (PDA). APCZ showed promising GSH-depleting ability and near-infrared (NIR)-II photothermal performance for combined cancer therapy. Once internalized by 4T1 cells, the outer ZIF-8 was rapidly degraded to trigger the release of CuS nanoparticles (NPs), which could react with local GSH and sequentially hydrogen peroxide (H_2_O_2_) to form hydroxyl radical (·OH) for CDT. More importantly, the hyperthermia generated by APCZ upon 1064 nm laser excitation not only permitted NIR-II photothermal therapy (PTT) and promoted CDT, but also triggered the decomposition of AIPH to give toxic alkyl radical (·R) for oxygen-independent PDT. Besides, the PDA together with CuS greatly decreased the GSH level and resulted in significantly enhanced PDT/CDT in both normoxic and hypoxic conditions. The tumors could be completely eradicated after 14 days of treatment due to the prominent therapeutic effects of PTT/PDT/CDT. Additionally, the feasibility of APCZ as a photoacoustic (PA) imaging contrast agent was also demonstrated.

**Conclusions:**

The novel APCZ could realize the cooperative amplification effect of free radicals-based therapies by NIR-II light excitation and GSH consumption, and act as a contrast agent to improve PA imaging, holding tremendous potential for efficient diagnosis and treatment of deep-seated and hypoxic tumors.

**Graphic abstract:**

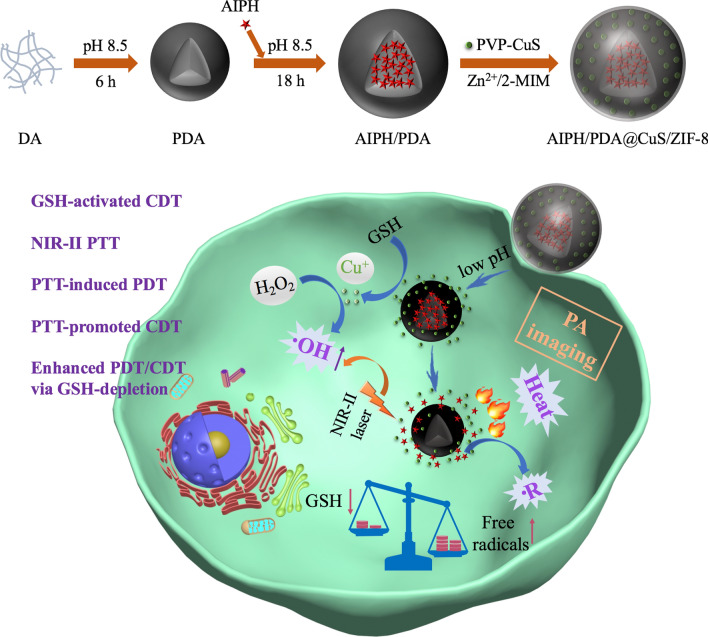

**Supplementary Information:**

The online version contains supplementary material available at 10.1186/s12951-021-01003-2.

## Background

Reactive oxygen species (ROS)-mediated photodynamic therapy (PDT) and chemodynamic therapy (CDT) have gained widespread attention in cancer theranostics [1–3]. However, hypoxia as a characteristic hallmark in solid tumors has been demonstrated to seriously hinder the performance of oxygen-dependent PDT. Besides, conventional PDT agents are usually activated by shorter wavelength lasers which are known to have only limited tissue penetration [4–6]. In view of these considerations, the use of free radical initiator, 2,2′-azobis[2-(2-imidazolin-2-yl)propane]-dihydrochloride (AIPH), has become a research hotspot as it allows the prompt production of toxic alkyl radical (·R) upon thermal stimulation and without the need of oxygen molecules [7, 8]. Therefore, the integration of AIPH with various photothermal agents (PTAs) onto a single nanoplatform is expected to offer a new modality for cancer therapy. To be specific, upon exposure to near-infrared (NIR) light, the PTAs rapidly convert the photo energy to hyperthermia for both photothermal therapy (PTT) and thermal decomposition of AIPH to give ·R for oxygen-independent PDT [9–12].

Unlike PDT, CDT is an emerging treatment method based on Fenton or Fenton-like reactions, which produces highly toxic hydroxyl radical (·OH) from hydrogen peroxide (H_2_O_2_) with transition metal catalysts [13–15]. Given the fact that tumor microenvironment (TME) has a relatively low pH and high H_2_O_2_ level, the production of ·OH in TME is more effective than that in the surrounding normal tissues, providing selectivity and low side effects for CDT. However, the overproduction of glutathione (GSH), a distinct feature in TME, can lead to increased resistance towards free radicals-induced cancer therapies owing to the potent scavenging effects [16–18]. Therefore, strategy to decrease the GSH level is pretty crucial for efficient PDT/CDT.

Copper sulfide (CuS), a popular p-type semiconductor material, has been extensively explored in biomedical applications [19–21]. Owing to the remarkable photothermal performance, CuS nanoparticles (NPs) have attracted considerable interest for cancer PTT in both NIR-I (700–1000 nm) and NIR-II (1000–1350 nm) bio-windows [22–24]. More importantly, Cu^2+^ is capable of depleting the intratumoral GSH and disrupting the redox homeostasis, and the resultant Cu^+^ could initiate a Fenton-like reaction to catalyze the production of ·OH from H_2_O_2_ under a wide range of pH conditions [25–27]. Considering the clinical applications and translation, a facile, mild and cost-effective synthetic method based on biocompatible and biodegradable nanomaterials is highly desirable.

**Scheme 1:**
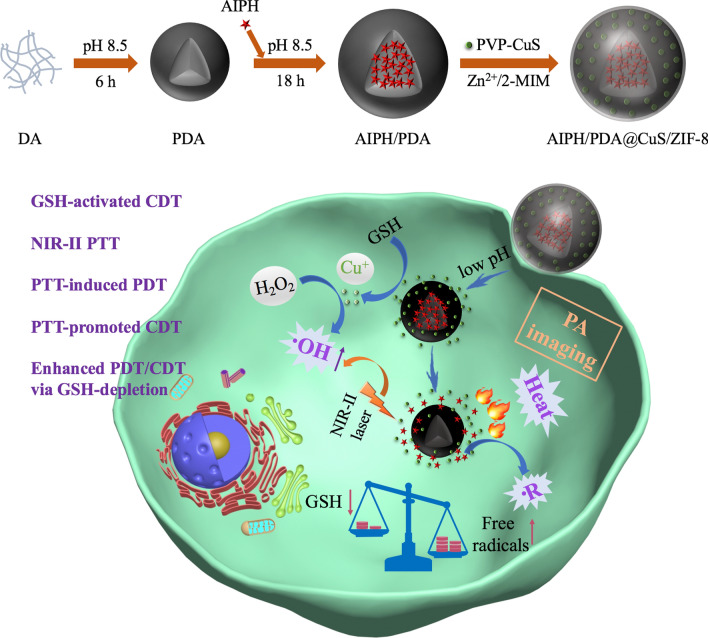
Schematic illustration of the preparation of APCZ and its application in PA imaging and synergistic NIR-II PTT/PDT/CDT

Herein, a smart free radical nanogenerator (AIPH/PDA@CuS/ZIF-8, denoted as APCZ) was constructed to address the above issues and achieve combined therapy (Scheme 1). The key of our design is the use of polydopamine (PDA) as core, which not only serves as a biocompatible and hydrophilic nanocarrier for AIPH but also facilitates the decoration of CuS NPs with the help of zeolitic imidazolate framework-8 (ZIF-8). The working mechanisms and advantages of the APCZ include: (1) the outer ZIF-8 can be rapidly degraded in acidic TME to release CuS NPs; (2) the CuS can react with the overproduced GSH to induce Cu^+^ generation; (3) the Cu^+^ can initiate an efficient Fenton-like reaction for CDT by catalyzing the production of ·OH from local H_2_O_2_; (4) the APCZ can utilize NIR-II light source for PTT; (5) the hyperthermia from PTT can trigger the decomposition of AIPH and produce toxic ·R for oxygen-independent PDT; (6) the photothermal effect also promotes the GSH depletion-triggered Fenton-like reaction; (7) the consumption of GSH by CuS and PDA greatly reduces the tumor antioxidant activity to improve the ·OH-/·R-induced therapeutic efficacy. In vitro and in vivo experiments evidenced these collaborative processes, and tumors were completely destroyed after 14 days of treatment via triple-modality therapy.

## Materials and methods

### Materials

Dopamine hydrochloride (98%), tris(hydroxymethyl)aminomethane (Tris, ≥ 99.8%), zinc nitrate hexahydrate (Zn(NO_3_)_2_·6H_2_O, 99.99%), 2-methylimidazole (2-MIM, 98%), 2,2′-azobis[2-(2-imidazolin-2-yl)propane] dihydrochloride (AIPH, > 98%), 2,2′-Azinobis(3-ethylbenzothiazoline-6-sulfonic acid ammonium salt) (ABTS, 98%), glutathione (GSH, 98%), 5,5′-dithiobis (2‐nitrobenzoic acid) (DTNB) and methylene blue (MB, ≥ 90%) were purchased from Aladdin Chemical Co. Ltd. Hydrogen peroxide (H_2_O_2_, 30%), hydrochloric acid (HCl, 36–38%), nitric acid (HNO_3_, 68%) and dimethylsulfoxide (DMSO, > 99.8%) were purchased from Sinopharm Chemical Reagent Co. Ltd., China. Copper chloride dihydrate (CuCl_2_·2H_2_O, ≥ 99.95%), sodium sulfide nonahydrate (Na_2_S·9H_2_O, ≥ 99.99%), polyvinyl pyrrolidone (PVP, Mw = 10,000), fetal bovine serum (FBS), Dulbecco’s Modified Eagle’s Medium (DMEM), Dulbecco’s phosphate-buffered saline (PBS), 2′,7′-Dichlorodihydrofluorescein diacetate (DCFH-DA, ≥ 97%), penicillin–streptomycin, trypsin, 3-(4,5-dimethylthiazol-2-yl)-2,5-diphenyltetrazolium bromide (MTT, 98%), 4′,6-diamidino-2-phenylindole dihydrochloride (DAPI), calcein acetoxymethyl ester (Calcein-AM), propidium iodide (PI), hematoxylin and eosin (H&E) staining kit and one step terminal deoxynucleotidyl transferase uridine triphosphate (dUTP) nick end-labeling (TUNEL) apoptosis assay kit were purchased form Sigma-Aldrich. All the reagents were used without further purification and aqueous solutions were prepared with deionized (DI) water.

### Synthesis of APCZ NPs

The AIPH-loaded PDA (AP) NPs were synthesized on the basis of a reported method with minor modifications [28]. Briefly, dopamine hydrochloride (25 mg) was first dissolved in Tris buffer (10 mM, 25 mL, pH 8.5) under vigorous stirring, during which the polymerization of dopamine (DA) was indicated by the darkening of the solution. After 6 h of reaction, AIPH (10 mg) was added and the reaction was allowed to proceed for another 18 h. The AP NPs were separated by centrifugation (18,000 rpm, 4 °C) and the supernatant was collected. The drug loading efficiency (DLE) and drug loading content (DLC) were calculated as (weight of AIPH in AP)/(weight of initial AIPH) × 100% and (weight of AIPH in AP)/(weight of PDA) × 100%, respectively. The obtained AP NPs were re-dispersed in DI H_2_O for further use.

The PVP-CuS NPs were obtained according to a reported method [29] but with the polyvinyl alcohol replaced by PVP. For the synthesis of APCZ NPs, the AP NPs (24 mg) was first dispersed in DI H_2_O (10 mL) with sonication, followed by addition of PVP-CuS dispersion (4 mg mL^−1^, 2 mL) and then aqueous Zn(NO_3_)_2_·6H_2_O solution (100 µg mL^−1^, 1485 µL) under vigorous stirring; aqueous 2-MIM solution (1 mg mL^−1^, 2870 µL) was then poured into the mixture and the reaction was kept for another 15 min. The resultant APCZ NPs were isolated by centrifugation (8000 rpm, 4 °C) and washed with DI H_2_O for several times. The APZ (AIPH/PDA@ZIF-8) and PCZ (PDA@CuS/ZIF-8) were synthesized in analogous fashion for various comparison studies.

### Characterization

Morphology was observed by transmission electron microscopy (TEM, FEI Tecnai F20). Hydrodynamic diameters and zeta potentials were investigated by dynamic light scattering (DLS, Autosize Loc-Fc-963, Malvern Instrument). Chemical composition was characterized by X-ray photoelectron spectroscopy (XPS). Crystal and chemical structures were examined by X-ray powder diffraction (XRD, D/MAX-IIIC, Japan) and Fourier transform infrared (FTIR, Perkin Elmer Spectrum 100) spectroscopies, respectively. Optical absorption spectra were obtained from an ultraviolet–visible–near infrared spectrophotometer (UV–vis–NIR, Evolution 300, Thermo Scientific, USA). Element contents in sample dispersions were analyzed by inductively coupled plasma atomic emission spectroscopy (ICP-AES, Thermo Scientific, USA).

### Photothermal effect measurements

The photothermal performance was investigated by exposing various concentrations (0–200 µg mL^−1^) of APCZ dispersions to a 1064 nm laser (1 W cm^−2^, 10 min). To evaluate the effect of power intensity on temperature variations, 200 µg mL^−1^ of APCZ aqueous dispersion was separately irradiated by a 1064 nm laser at different power intensities (0.5, 1 or 1.5 W cm^−2^) for 10 min. The changes in temperature as well as thermal images were recorded using an infrared thermal imager (B320V, Wuhan Guide Infrared Co., Ltd, China).

### ·R detection

ABTS was chosen to be the indicator for ·R generation as the ABTS free radical (ABTS^+^˙) showed characteristic optical absorption ranging from 400 to 900 nm. Briefly, aqueous mixture (2 mL) containing AIPH (20 µg mL^−1^) and ABTS (20 µg mL^−1^) were heated at 37 or 44 °C for 2, 4, and 6 h, respectively, and the UV–vis absorption spectra were recorded for analysis. To evaluate the impact of GSH on the ·R formation, aqueous mixture (2 mL) containing AIPH (20 µg mL^−1^), ABTS (20 µg mL^−1^), and GSH (0.5 mM) were heated at 37 or 44 °C for 6 h, respectively, followed by spectroscopic investigations. The results were compared with those obtained by aqueous mixture (2 mL) of APCZ (400 µg mL^−1^), ABTS (20 µg mL^−1^) and GSH (0.5 mM) heated at 44 °C (pH 5.0) for different time periods. To monitor the NIR-II light-induced ·R production, 2 mL aqueous mixture of APCZ (1 mg mL^−1^), ABTS (50 µg mL^−1^), and GSH (0.5 mM) was first irradiated by 1064 nm laser (1 W cm^−2^) for 10 min, and then incubated at pH 5.0 for 1, 2, 3, 4, 5, 6 h. The supernatant was collected and the absorption spectra were obtained for investigation.

### GSH depletion

To determine the GSH depletion by AP and CuS, DTNB (25 µM) was first mixed with different concentrations of GSH (12.5–50 µM) in PBS buffer (2 mL, pH 7.4) for 10 min to establish a standard curve of GSH by collecting the absorbance at 412 nm. To monitor the consumption of GSH by AP, different concentrations of AP (5–25 µg mL^−1^) dispersions were separately added to aqueous GSH (50 µM) solutions and allowed to stand for 12 h. DTNB (25 µM) was then added and the pH values of the mixtures were adjusted to 7.4. After incubation for another 10 min, the absorbance was recorded by a UV–vis–NIR spectrophotometer. The consumption of GSH by CuS was measured with the use of aqueous CuCl_2_ solution. Briefly, aqueous CuCl_2_ (50 µM) and GSH (50 µM) solutions were first mixed for different time periods, then DTNB (25 µM) was added and the pH values of the mixtures were adjusted to 7.4. After incubation for another 10 min, the absorbance for the mixtures were recorded for interpretation.

### ·OH detection

MB was used as the indicator for the detection of ·OH generated from Cu^+^-induced Fenton-like reaction. First of all, five groups were constructed for better understanding on the role of each component: MB only, MB + H_2_O_2_, MB + H_2_O_2_ + Cu^2+^, MB + H_2_O_2_ + Cu^2+^ + GSH and MB + H_2_O_2_ + Cu^2+^ + GSH + HCO_3_^−^ ([MB] = 10 µg mL^−1^, [H_2_O_2_] = 10 mM, [Cu^2+^] = 0.5 mM, [GSH] = 0.5 mM, [HCO_3_^−^] = 25 mM). After incubation for 1 h, the absorbance of the mixture was recorded by a UV–vis spectrophotometer. To further study the effect of reaction time and H_2_O_2_ or GSH concentration on the ·OH formation, the absorbances of the MB + H_2_O_2_ + Cu^2+^ + GSH + HCO_3_^−^ group ([MB] = 10 µg mL^−1^, [H_2_O_2_] = 10 mM, [Cu^2+^] = 0.5 mM, [GSH] = 0.5 mM, [HCO_3_^−^] = 25 mM) at specific time points (10–60 min) were recorded. Subsequently, the degradation of MB was investigated after treatment with different concentrations of H_2_O_2_ (0–10 mM) or GSH (0.25–8 mM), where the other parameters were kept unchanged. Additionally, PVP-CuS was used instead of Cu^2+^ to induce MB degradation. The absorbances of MB + H_2_O_2_ + PVP-CuS + GSH + HCO_3_^−^ group ([MB] = 10 µg mL^−1^, [H_2_O_2_] = 10 mM, [PVP-CuS] = 20 µg mL^−1^, [GSH] = 0.5 mM, [HCO_3_^−^] = 25 mM) at specific time points (5, 10 and 15 min) were recorded. To evaluate the photothermal effects on the Fenton-like reaction, the MB + H_2_O_2_ + PVP-CuS + GSH + HCO_3_^−^ group ([MB] = 10 µg mL^−1^, [H_2_O_2_] = 10 mM, [PVP-CuS] = 20 µg mL^−1^, [GSH] = 0.5 mM, [HCO_3_^−^] = 25 mM) was separately conducted at room temperature (25 °C), 37, 45 and 53 °C. After incubation for 5 min, the absorbances for the mixtures were recorded.

### Cellular uptake

Cellular uptake behavior was investigated using rhodamine B (RB)-loaded APCZ (RB-APCZ) NPs. First, APCZ (10 mg) and RB (2 mg) were dispersed in methanol (10 mL) under sonication, the resultant RB-APCZ NPs were collected by centrifugation (8000 rpm, 4 °C) after 24 h of gentle shaking. The RB-APCZ was then dispersed in fresh medium ([RB] = 5 µg mL^−1^) for cellular uptake experiment in accordance to our previous work [30]. After 1, 2 and 3 h of incubation, the cells were imaged by a confocal laser scanning microscope (CLSM, Leica SPE). Excitation wavelengths for DAPI and RB were 405 and 532 nm, respectively.

### Intracellular free radicals detection

DCFH-DA was chosen to be the indicator for the detection of intracellular free radicals production as its deacetylated product (non-fluorescent DCFH) could react with free radicals to form fluorescent 2′,7′-dichlorofluorescein (DCF). Under normoxic condition (21% O_2_), 4T1 cells (1 × 10^5^) were first seeded and cultured at 37 °C overnight, followed by incubating with (1) PBS, (2) blank, (3) AIPH, (4) APZ, (5) PCZ, (6) PCZ and (7) APCZ, respectively. After co-culturing for 4 h, group (2), (3), (6) and (7) were irradiated by 1064 nm laser (1 W cm^−1^, 10 min). Subsequently, these seven groups were treated with DAPI (10 µg mL^−1^) and DCFH-DA (10 µg mL^−1^) for another 25 min, the cells were then washed and imaged by CLSM. Likewise, group (5), (6) and (7) were conducted once more in a hypoxic atmosphere (1% O_2_). Excitation wavelengths for DAPI and DCF were 405 and 488 nm, respectively. To compare the free radicals generated from each group, the [AIPH] or/and [CuS] were set to be 12.6 µg mL^−1^ and 11.4 µg mL^−1^ respectively.

### Cytotoxicity measurement

The cytotoxicity of the samples towards 4T1 cells were first determined under normoxic condition. 4T1 cells (1 × 10^6^) were first seeded and cultured at 37 °C overnight, followed by incubating with (1) PBS, (2) blank, and different concentrations of (3) APZ, (4) PCZ, (5) PCZ and (6) APCZ, respectively. After co-culturing for 12 h, group (5) and (6) were irradiated by 1064 nm laser (1 W cm^−1^, 10 min) and then underwent incubation for another 12 h. Cell viabilities were determined using a standard MTT assay [31]. Likewise, group (4), (5) and (6) were conducted once more in a hypoxic atmosphere (1% O_2_). To visualize the cell-killing effects, all the aforementioned groups were co-stained with calcein-AM (2 µM) and PI (4 µM). After incubation for 30 min, the cells were washed and imaged by CLSM. Excitation wavelengths for calcein-AM and PI were 488 and 532 nm, respectively.

### Animal and tumor models

All animal experiments were approved by the Hong Kong Polytechnic University Animal Subjects Ethics Sub-Committee [Ref No. (20–208) in DH/HT&A/8/2/4 Pt.3]. Four-week-old female Balb/C nude mice were injected with 4T1 cells (1 × 10^6^) at the right hind legs to obtain subcutaneous tumor models. Animal experiments were conducted with an initial tumor size of around 100 mm^3^.

### In vitro and in vivo photoacoustic (PA) imaging

For in vitro PA imaging, APCZ dispersions with various concentrations (0–1 mg mL^−1^) were first prepared and the PA signals were then determined under a 970 nm laser treatment (Fujifilm Visual Sonics Vevo LAZR PA imaging system). For in vivo PA imaging, APCZ dispersion (20 mg kg^−1^) was first intravenously injected into the 4T1 tumor-bearing mouse and the PA signals were recorded at specific time points (0, 2, 5, 10, 16 and 24 h).

### In vivo combined therapy

To evaluate the in vivo anti-tumor effects, 4T1 tumor-bearing mice were randomized into six groups (n = 4 per group): (1) PBS; (2) 1064 nm Laser; (3) APZ; (4) APCZ; (5) PCZ + Laser; (6) PCZ + Laser. Photothermal-related experiments were conducted with 1064 nm laser (1 W cm^−1^, 10 min) after injection of the sample dispersions (20 mg kg^−1^) for 10 h. In vivo photothermal performance of PCZ and APCZ were studied via recording the temperature changes of the tumor site and a PBS-treated group was set as control. Subsequently, the changes of tumor volume and body weight in each group were measured every 2 days for 14 days to assess the therapeutic efficacy. After the 2-week treatment period, mice were sacrificed and the tumor sections and major organs (heart, liver, spleen, lung, and kidney) were collected to estimate the tissue damage by H&E and TUNEL staining assay.

### Blood circulation and biodistribution

To study the blood circulation of injected APCZ dispersion, blood samples were acquired at specific time points, followed by measuring the concentrations of Zn^2+^ through ICP-AES. For further evaluation on the biodistribution, tumor sections and major organs were first collected after 24 or 48 h post-injection of APCZ dispersion and then treated with aqua regia to determine the Zn^2+^ concentrations.

### Statistical analysis

Data are expressed as the mean ± standard deviation (SD). Statistical analysis was carried out by one-way ANOVA test with SPSS software (version 17.0, IBM Inc., Chicago, IL, USA). Statistical significance was set at *p < 0.05, **p < 0.01, ***p < 0.001.

## Results and discussion

### Design, synthesis and characterization of APCZ

PDA, a nature-inspired biopolymer, is recognized as a new class of biomaterials for biomedical applications. In addition to being a popular photothermal agent for PTT, the abundant aromatic rings and functional groups make it possible to load chemical drugs and carry out further chemical modifications [32–37]. On the other hand, ZIF-8 with intrinsic pore structures, excellent pH-responsive ability and low cytotoxicity, has been demonstrated to be an intelligent nanocarrier to delivery molecular drugs, diverse enzymes and nano-sized particles [38–41]. Taking full advantage of these merits, the APCZ NPs could be easily synthesized in situ by coating CuS-encapsulated ZIF-8 on the surface of AP NPs. Briefly, PDA was first used to load AIPH after 6 h of polymerization to obtain AP, which could further be functionalized with Zn^2+^ ions and CuS; aqueous 2-MIM solution was then added to induce the formation of ZIF-8 layer with simultaneous embedment of CuS. Since TME has a lower pH than normal tissues, the pH-responsive ZIF-8 coating is able to prevent the premature leakage of both CuS and AIPH, thus ensuring its biosafety. Moreover, the CuS embedded in the ZIF-8 can endow remarkably enhanced NIR-II absorption for PTT as well as GSH-depleting capability for the APCZ to provide free radicals-mediated therapies.

**Fig. 1:**
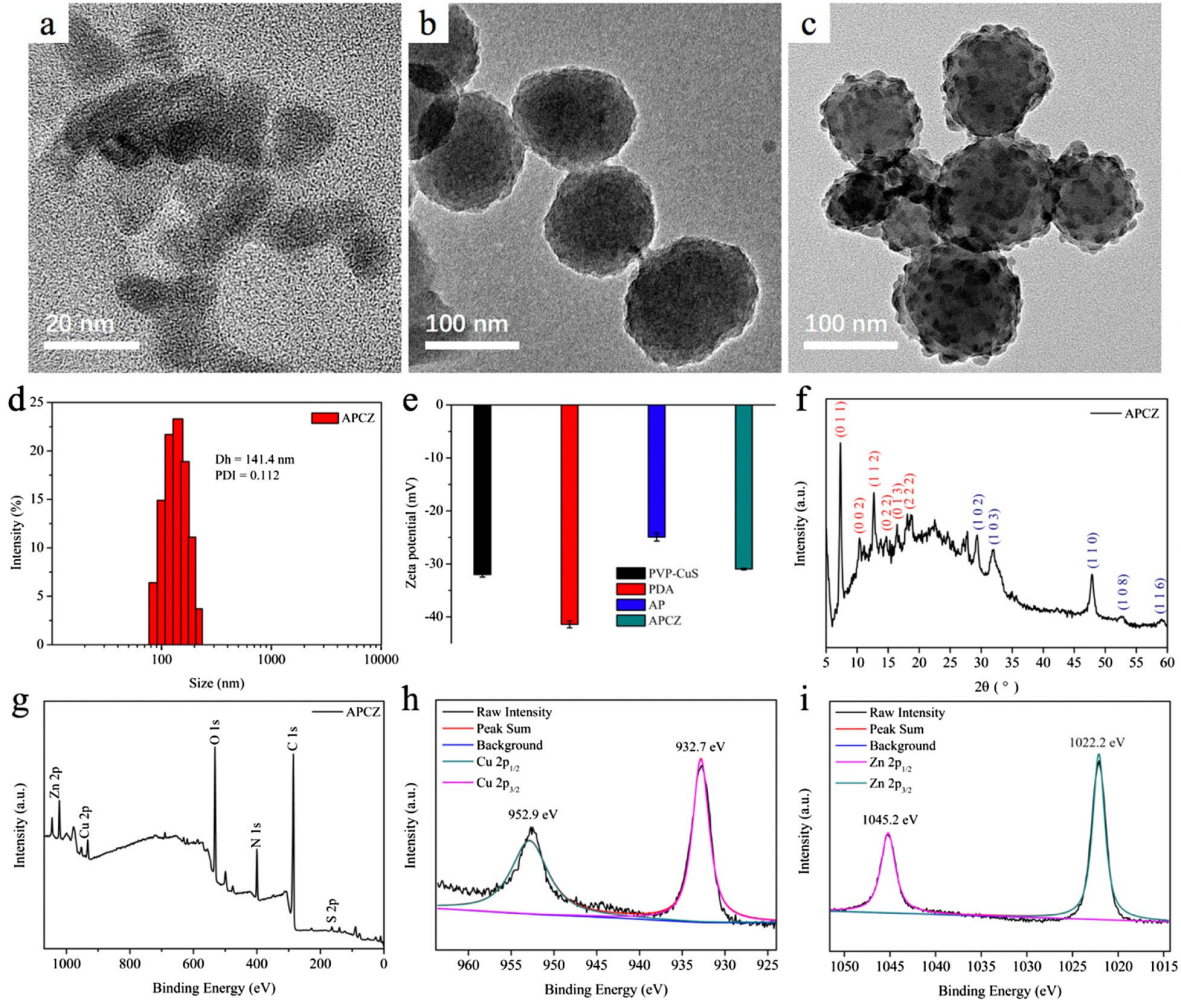
TEM images of **a** PVP-CuS, **b** AP and **c** APCZ. **d** Hydrodynamic diameter and size distribution of APCZ. **e** Zeta potentials of aqueous PVP-CuS, PDA, AP and APCZ dispersions. Data shown as mean ± SD, n = 3 per treatment. **f** XRD pattern of APCZ NPs. XPS spectra of **g** APCZ, **h** Cu 2p and **i** Zn 2p

**Fig. 2:**
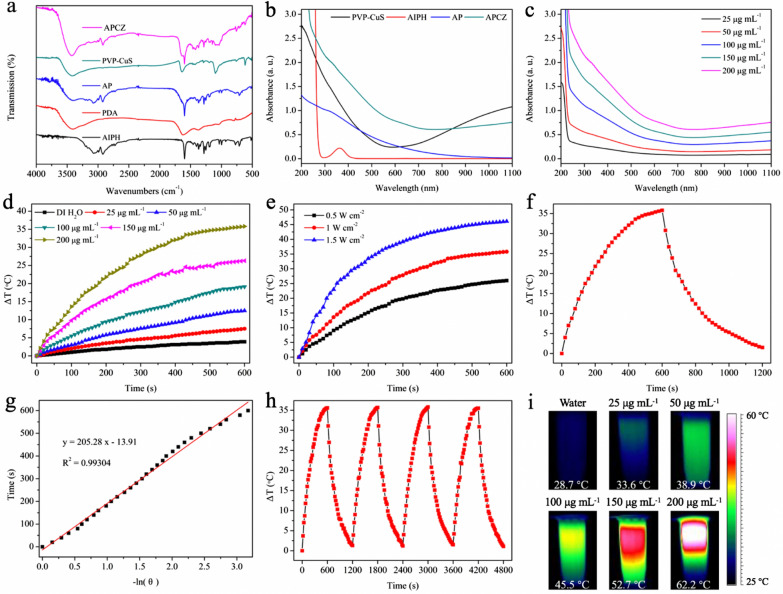
**a** FTIR spectra of AIPH, PDA, AP, PVP-CuS and APCZ. **b** UV–vis–NIR spectra of PVP-CuS, AIPH, AP and APCZ in DI water. **c** UV–vis–NIR spectra of aqueous APCZ dispersions at different concentrations. **d** Photothermal curves of aqueous APCZ dispersions at different concentrations exposed to a 1064 nm laser (1.0 W cm^−2^, 10 min). **e** Photothermal curves of aqueous APCZ dispersion (200 µg mL^−1^) exposed to a 1064 nm laser with different power densities (0.5, 1.0 and 1.5 W cm^−2^) for 10 min. **f** Photothermal response of aqueous APCZ dispersion (200 µg mL^−1^) with (first 10 min) and without (next 10 min) exposure to a 1064 nm laser (1.0 W cm^−2^). **g** Linear fitting for time and − ln θ. **h** Photothermal response of aqueous APCZ dispersion (200 µg mL^−1^) for four successive on/off cycles under 1064 nm laser (1 W cm^−2^, 10 min) irradiation. **i** Corresponding infrared thermal images for aqueous APCZ dispersions in **d** after laser irradiation for 10 min

TEM images showed that the PVP-CuS and AP NPs were uniform and monodispersed with average sizes of 12 nm and 103 nm, respectively (Fig. 1a, b). The APCZ NPs had an increased diameter (127 nm) due to the ZIF-8 coating and the embedded CuS NPs could also be clearly observed (Fig. 1c). DLS measurement showed that the average hydrodynamic diameter (Dh) and zeta potential of APCZ were 141.4 nm and − 30.9 mV, respectively. The Dh was larger than the TEM-determined size as the APCZ swelled in water, and the low polydispersity index (PDI) revealed a narrow size distribution (Fig. 1d, e). In addition, the negligible changes of size and zeta potential during the 14-day period of dialysis indicated good physiological stability of APCZ (Additional file 1: Figure S1). The crystalline structure of APCZ was analyzed by XRD (Fig. 1f). The diffraction peaks at 2θ = 7.3°, 10.5°, 12.7°, 14.6°, 16.4° and 18.1° were ascribed to the (0 1 1), (0 0 2), (1 1 2), (0 2 2), (0 1 3) and (2 2 2) planes of ZIF-8, while the peaks of CuS consistent with JCPDS card number 18-0802 were also observed [42, 43]. The XPS pattern of APCZ exhibited all the characteristic peaks of C 1s, N 1s, O 1s, Zn 2p, Cu 2p and S 2p, indicating the existence of each component. The peaks of Cu 2p (952.9 and 932.7 eV) and Zn 2p (1045.2 and 1022.2 eV) could be assigned to Cu^2+^ and Zn^2+^ (Fig. 1g–i). FTIR spectroscopy was utilized to study the chemical structure of the samples. As shown in Fig. 2a, the broad absorption peak around 3425 cm^−1^ could be assigned to the stretching vibration of O–H and N–H, and the typical indole-related peak at 1615 cm^−1^ could be attributed to the PDA (red curve). After loading with AIPH, the spectrum of AP presented the characteristic peaks of both AIPH and PDA. Besides, the peaks at 1640 and 620 cm^−1^ on the green curve were originated from the C=O in PVP and Cu–S in CuS respectively, indicating that the CuS was modified by PVP [44]. Additionally, those characteristic peaks were all found in the pink curve, suggesting that the APCZ NPs were successfully synthesized.

The UV–vis–NIR spectrum of APCZ showed enhanced optical absorption ranging from NIR-I to NIR-II region due to the PDA and CuS NPs, whereas the typical peak of AIPH was overlapped in the UV range (Fig. 2b, c). The DLE and DLC of AIPH were determined to be 49.8% and 20.75% respectively (Additional file 1: Figures S2, S3) [45]. The contents of AIPH, PDA, ZIF-8 and PVP-CuS in APCZ were calculated to be 6.3%, 24.08%, 58.23% and 11.39% respectively, while the mass ratio between AIPH and CuS was about 1.106:1. For the evaluation of NIR-II photothermal performance, APCZ dispersions of various concentrations (0–200 µg mL^−1^) were continuously exposed to a 1064 nm laser, and the temperature increases were found to be APCZ concentration- and irradiation duration-dependent. As shown in Fig. 2d, the APCZ dispersion (200 µg mL^−1^) had a temperature increase of 35.8 °C after 10 min of laser irradiation (1064 nm, 1 W cm^−2^), whereas only a slight temperature increase (3.9 °C) was found in the DI H_2_O group; the infrared thermal images of these samples just after the 10-min irradiation were depicted in Fig. 2i. By tuning the laser power intensity, the temperature increment of the aqueous APCZ dispersion (200 µg mL^−1^) could be changed from 26 °C (0.5 W cm^−2^) to 46.1 °C (1.5 W cm^−2^) (Fig. 2e). These results signified that APCZ was able to efficiently absorb 1064 nm light and transfer it into hyperthermia. The photothermal conversion efficiency (*η*) of APCZ NPs upon 1064 nm irradiation was calculated to be 28.05% according to the reported method (Fig. 2f, g) [46]. Moreover, the photothermal stability of APCZ NPs was studied via recording the temperature profiles of aqueous APCZ dispersion under four cycles of heating/cooling process. As shown in Fig. 2h, no obvious attenuation was found in every single test cycle, revealing the robustness of APCZ for NIR-II PTT. Furthermore, to clarify the contribution of the PDA and PVP-CuS NPs in the APCZ-induced photothermal conversion phenomenon, the photothermal performance of equivalent concentrations of PDA (48.16 µg mL^−1^) and PVP-CuS (22.78 µg mL^−1^) under the same conditions were investigated. After irradiation by a 1064 nm laser (1 W cm^−2^) for 10 min, the temperature increases of the PDA and PVP-CuS dispersions were found to be 13.6 and 22.1 °C respectively (Additional file 1: Figure S4).

More interestingly, NIR-II laser exposure not only endows APCZ NPs with satisfying photothermal performance for PTT but also the power to produce free radicals from the loaded AIPH. As GSH possesses potent free radicals-scavenging effects, the ability for APCZ to act as an effective GSH-depleting agent was first investigated by DTNB. As shown in Additional file 1: Figures S5, S6, DTNB possesses a characteristic peak around 325 nm while a new absorption at 412 nm could be observed after reaction with GSH. When incubating AP with GSH, the absorption peaks at 412 nm decreased with increasing AP concentration, and nearly 50 µM of GSH could be completely eliminated by 20 µg mL^−1^ of AP dispersion after 12 h (Additional file 1: Figure S7). This process could be attributed to the Schiff base/Michael addition reaction between GSH and the quinone groups of PDA [47, 48]. The optical absorbance of AP/GSH mixtures under different pH values were also recorded (Additional file 1: Figure S8). After GSH treatment, the absorbance of the mixture was obviously weakened (incubation for 12 h), and the further decrease in absorption at pH 5.0 revealed the feasibility of GSH consumption by AP in TME. Subsequently, the GSH-depleting ability of CuS was investigated by incubating CuCl_2_ and GSH for different time periods, and only 15.3% of GSH remained after 6 h of reaction according to the standard curve (Additional file 1: Figure S9). The degradation behaviors of CuS by GSH solutions were investigated by incubating PVP-CuS (25 µg mL^−1^) with different concentrations of GSH (1, 2, 4 and 10 mM) for 6 h and measuring the optical absorptions of the resultant mixtures (a total volume of 2 mL). As shown in Additional file 1: Figure S10, the gradual decrease of NIR absorption with the increased GSH concentration revealed the degradation of CuS by GSH. Meanwhile, the color changes of the mixtures also evidenced that PVP-CuS did in fact react with GSH (Additional file 1: Figure S11). Subsequently, the supernatants were collected to measure the Cu ions level by ICP−AES (Additional file 1: Figure S12). The morphologies of the PVP-CuS (25 µg mL^−1^) after reacting with GSH (10 mM) for 24 h were also investigated (Additional file 1: Figure S13); the disappearance of the uniform and monodisperse PVP-CuS NPs further confirmed that the CuS was capable of reacting with GSH. These data evidenced that our APCZ was able to reinforce GSH-consumption for enhanced free radicals-induced therapies. Owing to these TME-sensitive capabilities, the drug release behaviors of APCZ were then studied (Additional file 1: Figure S14). Negligible AIPH release was observed in both the pH 7.4 group and pH 7.4 + GSH group due to the pH-responsiveness of ZIF-8. However, the cumulative release of AIPH in acid-treated group (pH 5.0) could reach up to 37.32% within 24 h. Moreover, the drug release was accelerated in the pH 5.0 + GSH group and about 53.87% of the incorporated AIPH was released after 24 h. These results were further supported by the Cu ions release profiles with different treatments, revealing the great potential of APCZ as an ideal drug delivery system (Additional file 1: Figure S15).

**Fig. 3:**
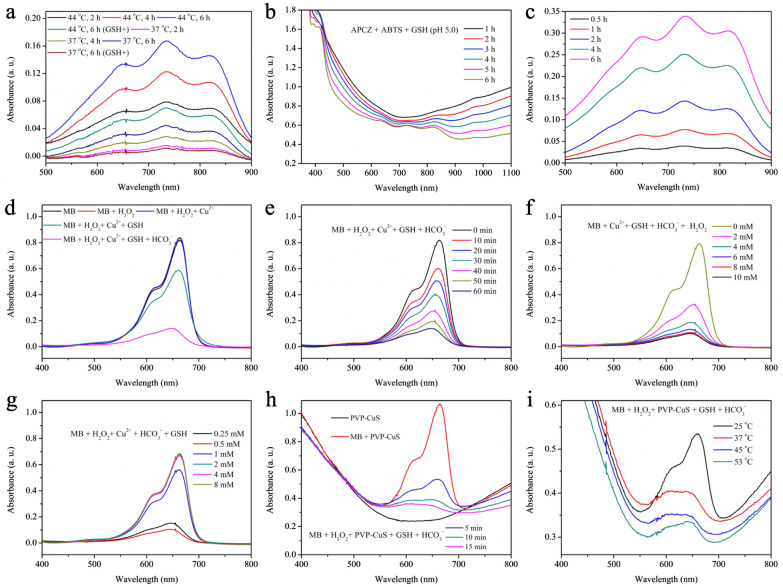
**a** Generation of ABTS^+^˙ in AIPH + ABTS and AIPH + ABTS + GSH at 37 or 44 °C for different time periods. [AIPH] = 20 µg mL^−1^, [ABTS] = 20 µg mL^−1^, [GSH] = 0.5 mM. **b** Generation of ABTS^+^˙ in APCZ + ABTS + GSH (pH 5.0) at 44 °C for different time periods. [APCZ] = 400 µg mL^−1^, [ABTS] = 20 µg mL^−1^, [GSH] = 0.5 mM. **c** The generation of ABTS^+^˙ in the APCZ + ABTS + GSH (pH 5.0) + Laser group. The APCZ + ABTS + GSH mixture was first exposed to 1064 nm laser (1.0 W cm^−2^, 10 min) and then incubated at pH 5.0 for different time periods. [APCZ] = 1 mg mL^−1^, [ABTS] = 50 µg mL^−1^, [GSH] = 1 mM. **d** The degradation of MB under different conditions for 60 min. [MB] = 10 µg mL^−1^, [H_2_O_2_] = 10 mM, [Cu^2+^] = 0.5 mM, [GSH] = 0.5 mM, [HCO_3_^−^] = 25 mM. **e** The degradation of MB as a function of time (10, 20, 30, 40, 50, 60 min). [MB] = 10 µg mL^−1^, [H_2_O_2_] = 10 mM, [Cu^2+^] = 0.5 mM, [GSH] = 0.5 mM, [HCO_3_^−^] = 25 mM. **f** The degradation of MB as a function of H_2_O_2_ concentration (0, 2, 4, 6, 8 and 10 mM) for 60 min. [MB] = 10 µg mL^−1^, [Cu^2+^] = 0.5 mM, [GSH] = 0.5 mM, [HCO_3_^−^] = 25 mM. **g** The degradation of MB in the presence of GSH (0.25, 0.5, 1, 2, 4 and 8 mM) for 60 min. [MB] = 10 µg mL^−1^, [H_2_O_2_] = 10 mM, [Cu^2+^] = 0.5 mM, [HCO_3_^−^] = 25 mM. **h** The degradation of MB by PVP-CuS-induced Fenton-like reaction for 5, 10 and 15 min. [MB] = 10 µg mL^−1^, [H_2_O_2_] = 10 mM, [PVP-CuS] = 20 µg mL^−1^, [GSH] = 0.5 mM, [HCO_3_^−^] = 25 mM. (i) The degradation of MB by PVP-CuS-induced Fenton-like reaction at room temperature (25 °C), 37, 45 and 53 °C for 5 min. [MB] = 10 µg mL^−1^, [H_2_O_2_] = 10 mM, [PVP-CuS] = 20 µg mL^−1^, [GSH] = 0.5 mM, [HCO_3_^−^] = 25 mM

The heat-induced AIPH decomposition was investigated at 37 and 44 °C. The generated ·R could react with ABTS to produce ABTS^+^˙, giving typical absorption peaks ranging from 500 nm to 900 nm. As shown in Fig. 3a, more ·R was generated at 44 °C than that at 37 °C (incubation for 6 h), suggesting that the decomposition of AIPH could be speeded up by increasing temperature. With GSH simultaneously treated, less ABTS^+^˙ was formed because of its free radicals-scavenging effects. Besides, the absorption peaks of ABTS^+^˙ were hardly found in the APCZ + GSH + ABTS group even at 44 °C due to the limited diffusion of ·R through the ZIF-8 shell, while the slight absorbance decrease from UV to NIR region could be attributed to the partial degradation of CuS by GSH (Additional file 1: Figure S16); when performed at pH = 5.0, characteristic absorption bands for ABTS^+^˙ was observed with prolonged reaction times even though the APCZ NPs were gradually degraded by acid/GSH (Fig. 3b). To evaluate the ·R generation by NIR-II light, APCZ + GSH + ABTS mixture was first irradiated by 1064 nm laser (1 W cm^−2^) for 10 min and then incubated at pH 5.0 for different time periods. The supernatant was collected at each time point and measured by UV–vis–NIR spectroscopy. As shown in Fig. 3c, typical absorption peaks of ABTS^+^˙ were observed and more ·R was released with extended time. The NIR laser-stimulated production and low pH-triggered release of ·R ensured the therapeutic selectivity of APCZ towards tumors.

On the other hand, CuS is capable of reacting with GSH to generate Cu^+^, which could initiate an efficient Fenton-like reaction for CDT by catalyzing the production of ·OH using local H_2_O_2_ as feedstock. Using MB as the indicator for ·OH formation, the pure MB, MB + H_2_O_2_ and MB + H_2_O_2_ + Cu^2+^ groups displayed negligible degradation of MB (incubation for 1 h), while obvious decrease of MB absorption was observed after GSH addition (Fig. 3d). More importantly, the accelerated MB discoloration in the MB + H_2_O_2_ + Cu^2+^ + GSH + HCO_3_^−^ group revealed the vital role of HCO_3_^−^ in Cu^+^-mediated Fenton-like reaction [49, 50]. Moreover, Fig. 3e, f indicated that the generation of ·OH exhibited time- and H_2_O_2_ concentration-dependent manners. The impact of GSH level towards ·OH production was also evaluated. While a small amount of GSH could facilitate the Cu^2+^-mediated Fenton-like reaction, excess GSH severely restricted the ·OH generation due to the scavenging effects (Fig. 3g). For the CuS NPs groups, the optical absorption showed a rapid downward trend and most of the MB was degraded within 5 min (Fig. 3h). Additionally, we found that the GSH depletion-triggered Fenton-like reaction could be boosted by increasing temperature, and much less MB remained when incubating the MB + H_2_O_2_ + PVP-CuS + GSH + HCO_3_^−^ at 53 °C (Fig. 3i). These results indicated the outstanding ·OH-generating ability of APCZ that was expected to be promoted by NIR-II laser.

**Fig. 4:**
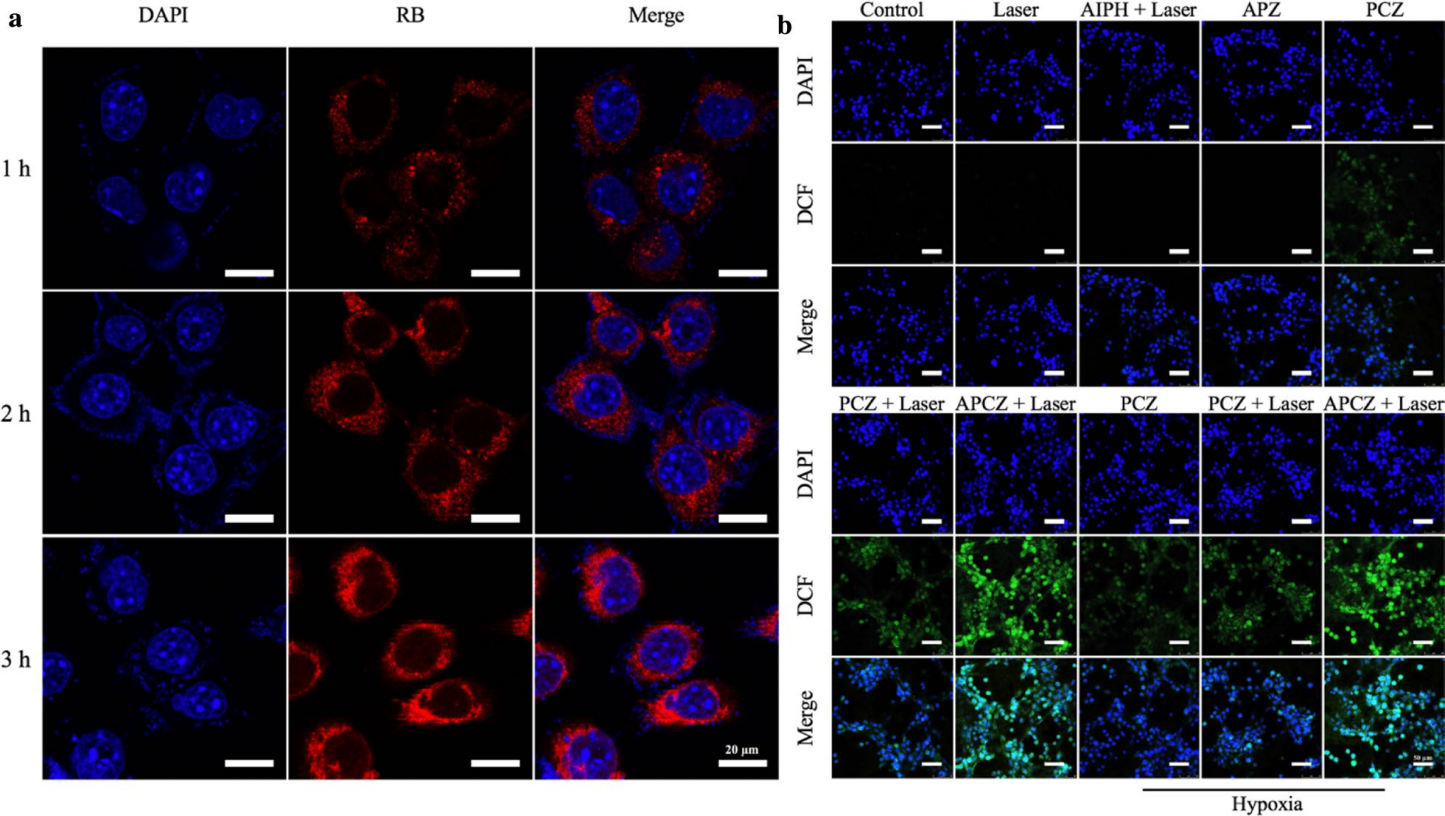
**a** Fluorescence images of 4T1 cells incubated with RB-APCZ ([RB] = 5 µg mL^−1^) for 1, 2 and 3 h. Scale bar = 20 μm. **b** Detection of intracellular ROS generation by DCFH-DA in both normoxic and hypoxic conditions. [AIPH] = 12.6 µg mL^−1^, [CuS] = 11.4 µg mL^−1^, scale bar = 50 μm

To assess the cellular uptake behavior, RB-APCZ NPs were separately incubated with 4T1 cells for 1, 2 and 3 h (Fig. 4a). Vivid red fluorescence was detected and the fluorescence intensity was significantly strengthened as incubation time increased, indicating the effective internalization of APCZ. Subsequently, the intracellular free radicals generation was evaluated in both normoxic and hypoxic conditions. A commonly used indicator DCFH-DA was chosen since it could be oxidized to fluorescent DCF in 4T1 cells by free radicals. As shown in Fig. 4b, negligible free radicals were formed in the Control, Laser, AIPH + Laser and APZ groups. In contrast, the PCZ-, PCZ + Laser- and APCZ + Laser-treated 4T1 cells displayed green fluorescence with different intensities. The weak DCF fluorescence presented in the PCZ group was due to the Cu^+^-induced Fenton-like reaction. Moreover, the 4T1 cells treated with PCZ exhibited improved green fluorescence upon 1064 nm laser exposure, revealing the photothermal-enhanced ·OH generating effect. Furthermore, the strongest fluorescence of DCF was noticed in the APCZ + Laser group as expected since the hyperthermia could also induce the decomposition of AIPH to produce ·R. Notably, the generation of free radicals with different treatments showed little difference under both normoxic or hypoxic conditions. These results suggested the great promise of our APCZ as a hypoxia-irrelevant free radical nanogenerator for highly efficient cancer therapy.

**Fig. 5:**
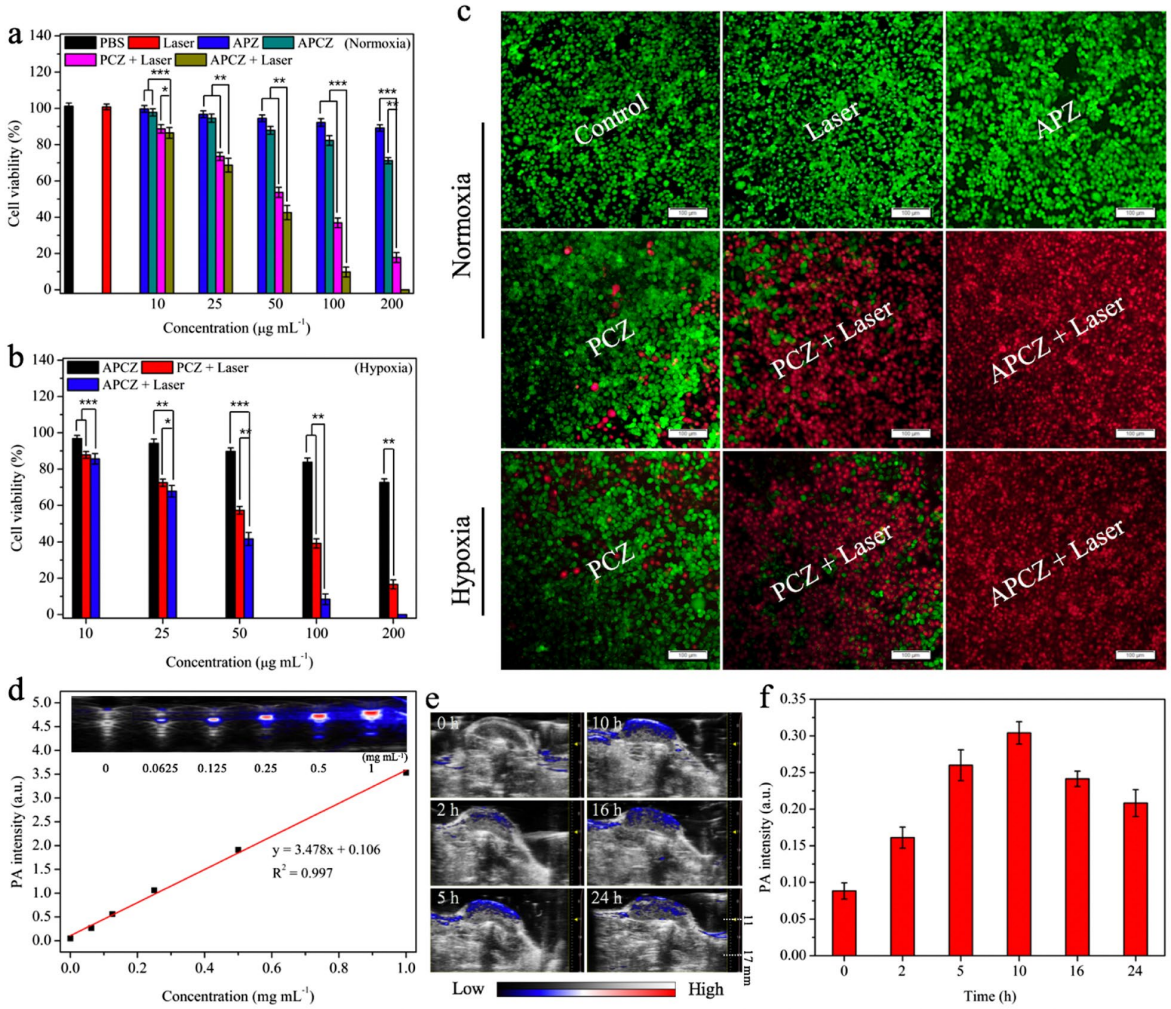
Cell viabilities of 4T1 cells after different treatments in normoxic (**a**) and hypoxic (**b**) conditions. Data shown as mean ± SD, n = 3 per treatment. Statistical significance was set at *p < 0.05, **p < 0.01, ***p < 0.001. **c** The calcein AM/PI co-stained images of 4T1 cells after different treatments in both normoxic and hypoxic conditions. Scale bar = 50 μm. **d** PA values as a function of APCZ concentration. Insert shows the corresponding in vitro PA images. **e** In vivo PA images of the tumor site after tail intravenous injection of APCZ dispersion for different time periods (0, 2, 5, 10, 16, 24 h). **f** Average PA values at tumor site corresponding to **e**. Data shown as mean ± SD, n = 3 per treatment

In view of the promising capabilities of NIR-II photothermal transduction and ·R-/·OH-generation, the in vitro anti-tumor performance of APCZ was investigated against 4T1 cells under different oxygen levels. The MTT assay experiments under normoxic condition showed that the PBS or laser treatment caused no apparent cytotoxicity towards 4T1 cells, and the cell viability in APZ group remained over 89% even at 200 µg mL^−1^ after 24 h incubation (Fig. 5a). However, dose-dependent cell-killing effects were observed in the PCZ group owing to single CDT. The PCZ + Laser group was found to be much more cytotoxic than the PCZ group owing to the NIR-II photothermal-enhanced CDT, and the cell viability could be decreased to 17.8% when the PCZ concentration was 200 µg mL^−1^; remarkably, the APCZ + Laser group exhibited more significant cytotoxicity and nearly no 4T1 cells survived at the same concentration because of the distinguished treatment outcome of the combined PTT/PDT/CDT. As expected, the cell viabilities of PCZ, PCZ + Laser, APCZ + laser groups under hypoxic condition also displayed similar results (Fig. 5b). In addition, the half maximal inhibitory concentration (IC_50_) values of different groups were calculated from the Prism dose-response curve (statistical program) by plotting the percentage of inhibition against the concentrations, showing consistent trends with the therapeutic effects. Under normoxic condition, the APCZ + Laser group had an IC_50_ value of 49.09 µg mL^−1^, which was much lower than that of the PCZ + Laser (59.02 µg mL^−1^) and PCZ (559.77 µg mL^−1^) groups. Likewise, the IC_50_ values of PCZ, PCZ + Laser and APCZ + Laser groups under hypoxic condition were calculated to be 560.12, 61.32, and 48.50 µg mL^−1^, respectively (Additional file 1: Figure S17 and Table S1). For better visualization of the live/dead cells, calcein AM and PI staining assay was used (Fig. 5c). The red fluorescence in the APCZ + Laser (normoxia or hypoxia) was the strongest among all the tested groups, further confirming the excellent efficacy of the triple-modality therapy. These results also demonstrated the oxygen-independent feature of the APCZ-based therapies for hypoxic tumors.

**Fig. 6:**
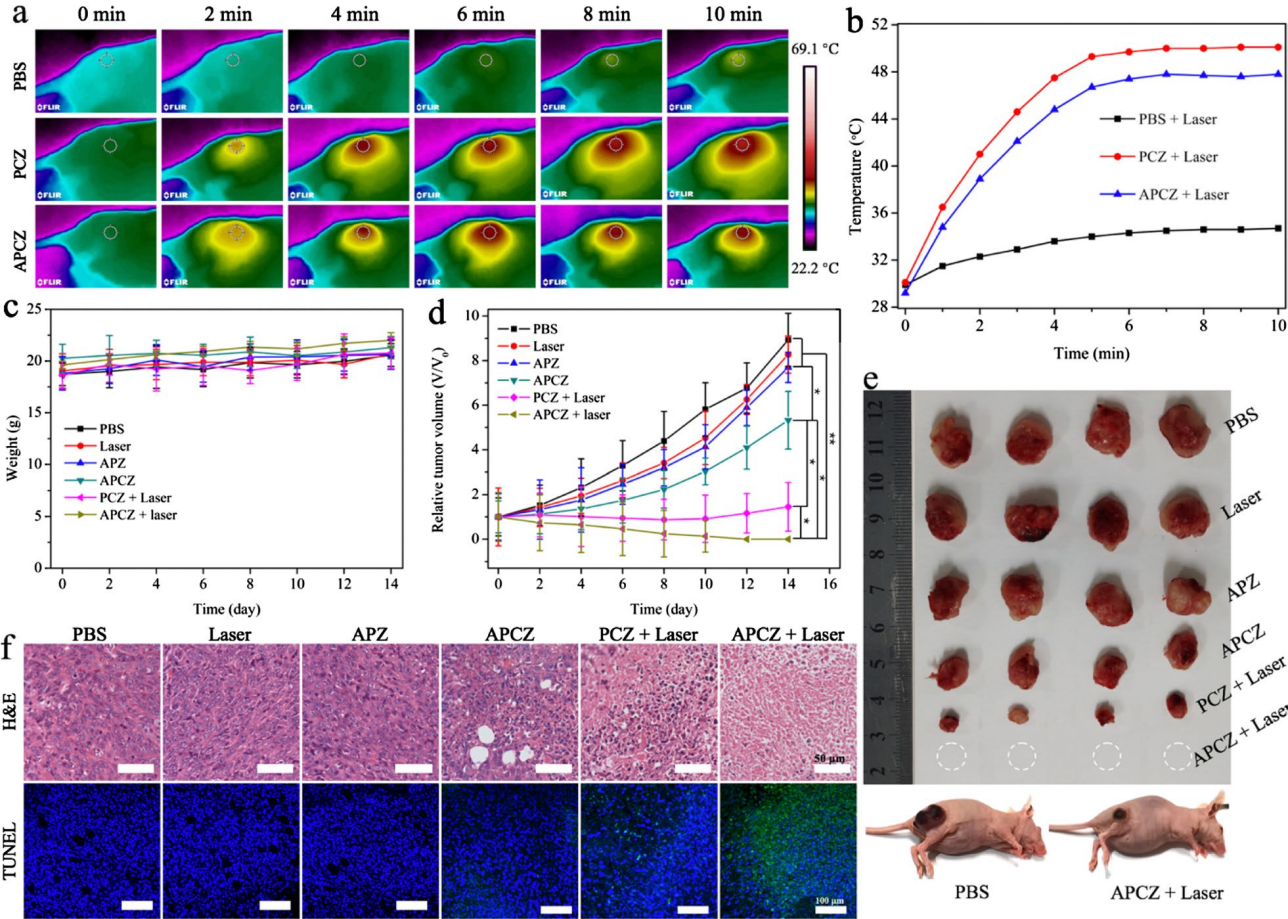
**a** Infrared thermal images and **b** temperature changes at the tumor site under 1064 nm laser irradiation for 2, 4, 6, 8 and 10 min. **c** The body weight changes and **d** the relative tumor growth curves in different groups. Data shown as mean ± SD, n = 4 per treatment. Statistical significance was set at *p < 0.05, **p < 0.01. **e** Digital images of tumors after different treatments and representative mice after treatment with PBS or APCZ + Laser. **f** Histological H&E and TUNEL staining tumor sections from different treatment groups. Scale bars = 50 and 100 μm, respectively

For cancer theranostics, imaging technology is often involved to detect and locate the tumor site as well as to guide the treatment process. Recently, high-resolution and noninvasive PA imaging has become one of the most popular imaging techniques [51, 52]. The broad and strong optical absorption from NIR-I to NIR-II enables APCZ to be a promising contrast agent for PA imaging. Obvious PA signals were observed in Fig. 5d, and the signal intensities presented a concentration-dependent manner under 970 nm laser irradiation. Subsequently, the in vivo PA imaging was carried out by intravenously injecting APCZ aqueous dispersion (20 mg kg^−1^) into 4T1 tumor-bearing mouse. The PA images of the tumor site were recorded at different time points (0, 2, 5, 10, 16, and 24 h) after injection. As shown in Fig. 5e, f, the PA signals exhibited a steady increase over time and reached a peak at 10 h post-injection because of the enhanced permeability and retention (EPR) effect [53, 54]. These results evidenced the effective accumulation of APCZ in tumor and 10 h was an ideal time point for PTT. As shown in Fig. 6a, b, in vivo photothermal effects of the APCZ and PCZ were confirmed by the impressive temperature increases (ΔT = 18.6 and 20 °C, respectively) at tumor site via 10 min of laser (1064 nm, 1 W cm^−2^) irradiation, while minimal temperature increment was found in the PBS-treated group. Besides, the blood circulation curve of as-prepared APCZ exhibited a double compartment pharmacokinetic model, and the plasma terminal half-life was calculated to be t_1/2_ = 1.60 h (Additional file 1: Figure S18a). The biodistribution of APCZ NPs in different organs was also investigated, the highest uptake of APCZ was in liver (~ 18.73% ID/g) at 24 h post-injection, followed by spleen (~ 15.08% ID/g) and tumor (~ 8.94% ID/g), while the content in heart, lung or kidney was at lower level (Additional file 1: Figure S18b).

To further study the in vivo anti-tumor efficacy, 4T1 tumor-bearing mice were randomized to six groups (n = 4 per group): (1) PBS; (2) Laser; (3) APZ; (4) APCZ; (5) PCZ + Laser; (6) APCZ + Laser. After 14 days of treatment, all groups presented insignificant decrease in body weight, implying the low systemic toxicity of the samples (Fig. 6c). Figure 6d, e gave the corresponding tumor volume changes during the therapies. It was clear that PBS, 1064 nm laser or APZ was not able to suppress the tumor growth. In contrast, the tumors in APCZ-treated groups were inhibited to a certain extent due to the CDT effect. Upon a 1064 nm laser irradiation, tumor growth was greatly restrained because of the cooperative PTT-enhanced CDT. Notably, the APCZ + Laser group achieved the strongest anti-tumor efficiency, which were reflected in the eradication of tumors after a 2-week treatment. Besides, some immune factors such as tumor necrosis factor-α (TNF-α) and interferon-γ (IFN-γ) for inhibiting tumor progression were increased after APCZ, PCZ + Laser and APCZ + Laser treatments for 7 days compared with those treated with PBS, Laser and APZ. Notably, the APCZ + Laser-based PTT/PDT/CDT group displayed the greatest enhancement, which was highly consistent with the therapeutic effects. These results suggested that our APCZ was also able to induce immunological stimulation effects in vivo (Additional file 1: Figure S19). Additionally, the tumor sections were collected and sliced for the H&E and TUNEL/DAPI staining assays (Fig. 6f). These data were consistent with the treatment outcome both in vitro and in vivo, in which the apoptotic cells in the APCZ + Laser group exhibited obvious nuclear contraction/cytoplasmic leakage and green fluorescence owing to the collaborative PTT/PDT/CDT. The ignorable tissue damage in the major organs also revealed the high biological safety of our APCZ NPs for promising clinical applications (Additional file 1: Figure S20).

## Conclusions

In summary, a novel TME and NIR II light-activated free radical nanogenerator APCZ was developed for cancer PA imaging guided PTT/PDT/CDT. Owing to the excellent pH responsiveness, the outer ZIF-8 layer could be rapidly degraded in TME to release CuS, which served as a self-reinforcing Fenton-like nanoagent for CDT by sequentially reacting with intratumoral GSH and H_2_O_2_. Besides, the APCZ displayed satisfying photothermal performance that not only permitted NIR-II PTT but also synergized with CDT as well as decomposed the AIPH to toxic ·R for oxygen-independent PDT. Notably, the depletion of GSH by PDA/CuS greatly decreased the tumor antioxidant activity and further led to enhanced ·R-/·OH-based therapies. Such a cooperative manner resulted in remarkable anti-tumor efficacy both in vitro (normoxia/hypoxia) and in vivo. Additionally, the APCZ was also demonstrated to act as an ideal PA imaging contrast agent, showing tremendous potential for hypoxia-irrelevant cancer theranostics.

## Supplementary Information

**Additional file 1: Figure S1.** (a) Hydrodynamic diameter of APCZ within 14-day dialysis in PBS buffer (pH 7.4). (b) Zeta potentials of aqueous APCZ dispersion before and after 14 day’s dialysis in PBS buffer (pH 7.4). Data shown as mean ± SD, n = 3 per treatment. **Figure S2.** (a) UV–vis absorption spectra of AIPH at various concentrations. (b) Standard curve of AIPH determined from (a) at 364 nm. **Figure S3.** UV–vis absorption spectra of AIPH before and after loading (solutions were diluted 5-fold for measurements). **Figure S4.** Photothermal curves of aqueous PDA (48.16 µg mL^−1^) and PVP-CuS (22.78 µg mL^−1^) dispersions exposed to a 1064 nm laser (1.0 W cm^−2^, 10 min). **Figure S5.** UV–vis absorption spectra of DTNB at various concentrations (10 −50 µM). **Figure S6.** (a) UV–vis absorption spectra of DTNB (25 µM) with various concentrations of GSH (12.5, 25, 37.5, and 50 µM). (b) Standard curve determined from (a) at 412 nm. **Figure S7.** Detection of GSH (50 µM) depletion by various concentrations of AP dispersions (5, 10, 15, 20 and 25 µg mL^−1^) after 12 h of reaction. [DTNB] = 25 µM. **Figure S8.** The degradation of AP in GSH, acid (pH 5.0) and acidic GSH (pH 5.0) for 12 h. [GSH] = 1 mM, [AP] = 100 µg mL^−1^. **Figure S9.** Detection of GSH (50 µM) depletion by aqueous CuCl_2_ (50 µM) solution for 10, 30, 60, 120, 180, 240 and 360 min, respectively. [DTNB] = 25 µM. **Figure S10.** UV–vis absorption spectra of PVP-CuS (25 µg mL^−1^) after incubation with various concentrations of GSH (0, 1, 2, 4 and 10 mM) for 6 h. **Figure S11.** Digital photos of PVP-CuS/GSH mixtures (separated by centrifugation and re-dispersed in 400 µL of DI H_2_O) after 6 h of reaction. [PVP-CuS] = 25 µg mL^−1^, [GSH] = (1) 0 mM, (2) 1 mM, (3) 2 mM, (4) 4 mM and (5) 10 mM. **Figure S12.** Relative Cu ions release from PVP-CuS/GSH mixtures after incubation for 6 h. [PVP-CuS] = 25 µg mL^−1^, [GSH] = 1, 2, 4 and 10 mM. **Figure S13.** TEM images of (a) PVP-CuS and (b-f) PVP-CuS/GSH mixtures after 24 h of reaction. [PVP-CuS] = 25 µg mL^−1^, [GSH] = 10 mM. **Figure S14.** Cumulative AIPH release profile of APCZ in PBS buffer of pH 7.4, pH 7.4 + GSH (10 mM), pH 5.0 and pH 5.0 + GSH (10 mM) for 24 h. Data shown as mean ± SD, n = 3 per treatment. **Figure S15.** Cumulative Cu ions release profile of APCZ in PBS buffer of pH 7.4, pH 7.4 + GSH (10 mM), pH 7.4 + GSH (10 mM) + Laser, pH 5.0, pH 5.0 + GSH (10 mM) and pH 5.0 + GSH (10 mM) + Laser. The orange arrows represented laser (1064 nm, 1.0 W cm^−2^) treatment, each time point was radiated for 10 min. Data shown as mean ± SD, n = 3 per treatment. **Figure S16.** UV–vis−NIR absorption spectra of APCZ and APCZ/GSH mixture after incubation in aqueous ABTS solution (44 °C) for 6 h. [APCZ] = 400 µg mL^−1^, [GSH] = 0.5 mM, [ABTS] = 20 µg mL^−1^. **Figure S17.** IC_50_ of PCZ group in normoxic condition calculated from MTT results by GraphPad Prism 8 software. **Figure S18.** (a) The blood clearance kinetics of APCZ after intravenously administration. (b) Biodistribution analysis of APCZ in 4T1 tumor bearing mice after the tail vein injection for 24 and 48 h. Data shown as mean ± SD, n = 3 per treatment. **Figure S19.** Standard curves of (a) mouse TNF-α and (b) mouse IFN-γ. O.D. means optical density (absorbance at 450 nm). (c) TNF-α and (d) IFN-γ levels in sera isolated from different groups after 7-day treatments. Data shown as mean ± SD, n = 3 per treatment. Statistical significance was set at *p < 0.05, **p < 0.01, ***p < 0.001. **Figure S20.** H&E staining images of major organs after different treatments. Scale bars = 50 μm. **Table S1.** IC_50_ of different groups calculated from MTT results by GraphPad Prism 8 software.

## Data Availability

All data analyzed during this study are included in this published article and its Additional file.

## Abbreviations

PDT: Photodynamic therapy
CDT: Chemodynamic therapy
GSH: Glutathione
APCZ: AIPH/PDA@CuS/ZIF-8
APZ: AIPH/PDA@ZIF-8
PCZ: PDA@CuS/ZIF-8
NIR: Near-infrared
CuS: Copper sulphide
ZIF-8: Zeolitic imidazolate framework-8
AIPH: 2,2′-Azobis[2-(2-imidazolin-2-yl)propane]-dihydrochloride
DA: Dopamine
PDA: Polydopamine
TME: Tumor microenvironment
NPs: Nanoparticles
H_2_O_2_: Hydrogen peroxide
·OH: Hydroxyl radical
PTT: Photothermal therapy
·R: Alkyl radical
PA: Photoacoustic
ROS: Reactive oxygen species
PTAs: Photothermal agents
Tris: Tris(hydroxymethyl)aminomethane
2-MIM: 2-Methylimidazole
ABTS: 2,2′-Azinobis(3-ethylbenzothiazoline-6-sulfonic acid ammonium salt)
DTNB: 5,5′‐Dithiobis (2‐nitrobenzoic acid)
MB: Methylene blue
HCl: Hydrochloric acid
HNO_3_: Nitric acid
DMSO: Dimethylsulfoxide
PVP: Polyvinyl pyrrolidone
FBS: Fetal bovine serum
DMEM: Dulbecco’s Modified Eagle’s Medium
PBS: Dulbecco’s phosphate-buffered saline
DCFH-DA: 2′,7′-Dichlorodihydrofluorescein diacetate
MTT: 3-(4,5-Dimethylthiazol-2-yl)-2,5-diphenyltetrazolium bromide
DAPI: 4′,6-Diamidino-2-phenylindole dihydrochloride
Calcein-AM: Calcein acetoxymethyl ester
PI: Propidium iodide
H&E: Hematoxylin and eosin
TUNEL: Terminal deoxynucleotidyl transferase uridine triphosphate (dUTP) nick end-labeling
DI: Deionized
AP: AIPH-loaded PDA
TEM: Transmission electron microscopy
DLS: Dynamic light scattering
XPS: X-ray photoelectron spectroscopy
XRD: X-ray powder diffraction
FTIR: Fourier transform infrared
UV–vis–NIR: Ultraviolet–visible–near infrared
ICP–AES: Inductively coupled plasma atomic emission spectroscopy
RB: Rhodamine B
DCF: 2′,7′-Dichlorofluorescein
SD: Standard deviation
Dh: Hydrodynamic diameter
PDI: Polydispersity index
ABTS^+^˙: ABTS free radical
EPR: Enhanced permeability and retention
TNF-α: Tumor necrosis factor-α
IFN-γ: Interferon gamma
