# Supplementary material for: NIR-II-driven and glutathione depletion-enhanced hypoxia-irrelevant free radical nanogenerator for combined cancer therapy
Source: J Nanobiotechnology. 2021 Sep 6;19:265. doi: 10.1186/s12951-021-01003-2 (PMC8420023; doi:10.1186/s12951-021-01003-2)
Supplement: Supplementary file 1 — Additional file 1: Figure S1. (a) Hydrodynamic diameter of APCZ within 14-day dialysis in PBS buffer (pH 7.4). (b) Zeta potentials of aqueous APCZ dispersion before and after 14 day’s dialysis in PBS buffer (pH 7.4). Data shown as mean ± SD, n = 3 per treatment. Figure S2. (a) UV–vis absorption spectra of AIPH at various concentrations. (b) Standard curve of AIPH determined from (a) at 364 nm. Figure S3. UV–vis absorption spectra of AIPH before and after loading (solutions were diluted 5-fold for measurements). Figure S4. Photothermal curves of aqueous PDA (48.16 µg mL−1) and PVP-CuS (22.78 µg mL−1) dispersions exposed to a 1064 nm laser (1.0 W cm−2, 10 min). Figure S5. UV–vis absorption spectra of DTNB at various concentrations (10 −50 µM). Figure S6. (a) UV–vis absorption spectra of DTNB (25 µM) with various concentrations of GSH (12.5, 25, 37.5, and 50 µM). (b) Standard curve determined from (a) at 412 nm. Figure S7. Detection of GSH (50 µM) depletion by various concentrations of AP dispersions (5, 10, 15, 20 and 25 µg mL−1) after 12 h of reaction. [DTNB] = 25 µM. Figure S8. The degradation of AP in GSH, acid (pH 5.0) and acidic GSH (pH 5.0) for 12 h. [GSH] = 1 mM, [AP] = 100 µg mL−1. Figure S9. Detection of GSH (50 µM) depletion by aqueous CuCl2 (50 µM) solution for 10, 30, 60, 120, 180, 240 and 360 min, respectively. [DTNB] = 25 µM. Figure S10. UV–vis absorption spectra of PVP-CuS (25 µg mL−1) after incubation with various concentrations of GSH (0, 1, 2, 4 and 10 mM) for 6 h. Figure S11. Digital photos of PVP-CuS/GSH mixtures (separated by centrifugation and re-dispersed in 400 µL of DI H2O) after 6 h of reaction. [PVP-CuS] = 25 µg mL−1, [GSH] = (1) 0 mM, (2) 1 mM, (3) 2 mM, (4) 4 mM and (5) 10 mM. Figure S12. Relative Cu ions release from PVP-CuS/GSH mixtures after incubation for 6 h. [PVP-CuS] = 25 µg mL−1, [GSH] = 1, 2, 4 and 10 mM. Figure S13. TEM images of (a) PVP-CuS and (b-f) PVP-CuS/GSH mixtures after 24 h of reaction. [PVP-CuS] = 25 µg mL−1, [GSH] = 10 [file 12951_2021_1003_MOESM1_ESM.docx]

Supplementary Material

NIR-II-driven and glutathione depletion-enhanced hypoxia-irrelevant free radical nanogenerator for combined cancer therapy

Li Zhang^1†^, Yadi Fan^2†^, Zhe Yang^1^, Mo Yang^2*^ and Chun-Yuen Wong^1,3*^

^1^ Department of Chemistry, City University of Hong Kong, Tat Chee Avenue, Kowloon, Hong Kong SAR

^2^ Department of Biomedical Engineering, The Hong Kong Polytechnic University, Hung Hom, Kowloon, Hong Kong SAR

^3^ State Key Laboratory of Terahertz and Millimeter Waves, City University of Hong Kong, Tat Chee Avenue, Kowloon, Hong Kong SAR

^*^Correspondence: acywong@cityu.edu.hk (C.-Y. Wong), mo.yang@polyu.edu.hk (M. Yang).

^†^Li Zhang and Yadi Fan contributed equally to this work.


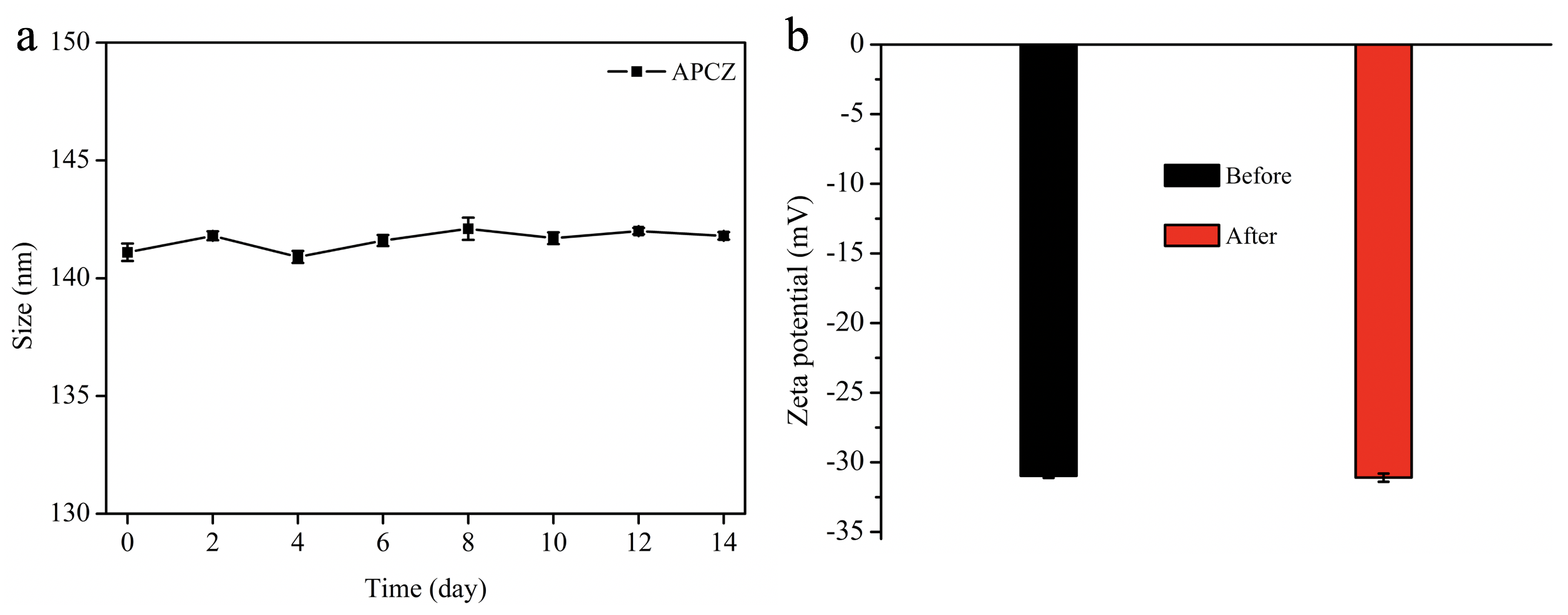


**Figure S1** (a) Hydrodynamic diameter of APCZ within 14-day dialysis in PBS buffer (pH 7.4). (b) Zeta potentials of aqueous APCZ dispersion before and after 14 day’s dialysis in PBS buffer (pH 7.4). Data shown as mean ± SD, n = 3 per treatment.


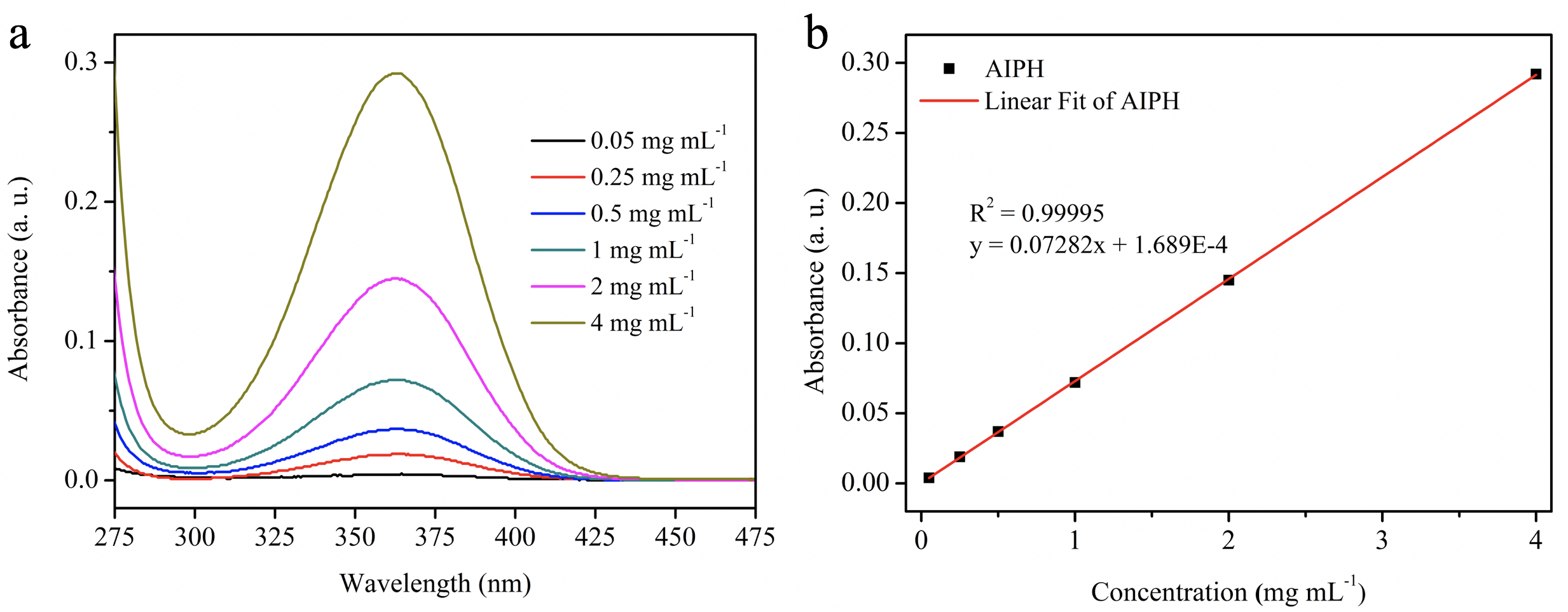


**Figure S2** (a) UV-vis absorption spectra of AIPH at various concentrations. (b) Standard curve of AIPH determined from (a) at 364 nm.


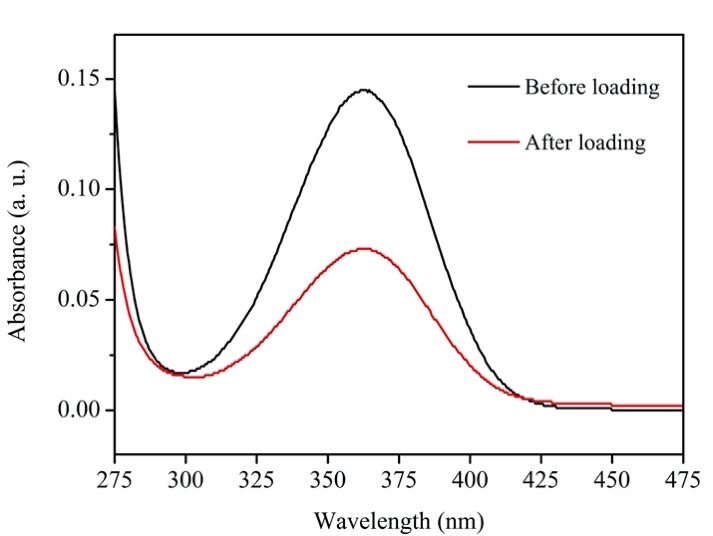


**Figure S3** UV-vis absorption spectra of AIPH before and after loading (solutions were diluted 5-fold for measurements).


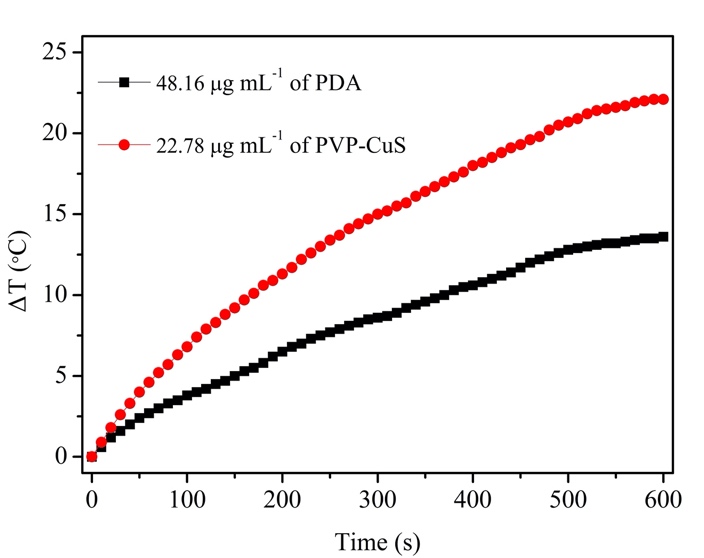


**Figure S4** Photothermal curves of aqueous PDA (48.16 μg mL^−1^) and PVP-CuS (22.78 μg mL^−1^) dispersions exposed to a 1064 nm laser (1.0 W cm^−2^, 10 min).


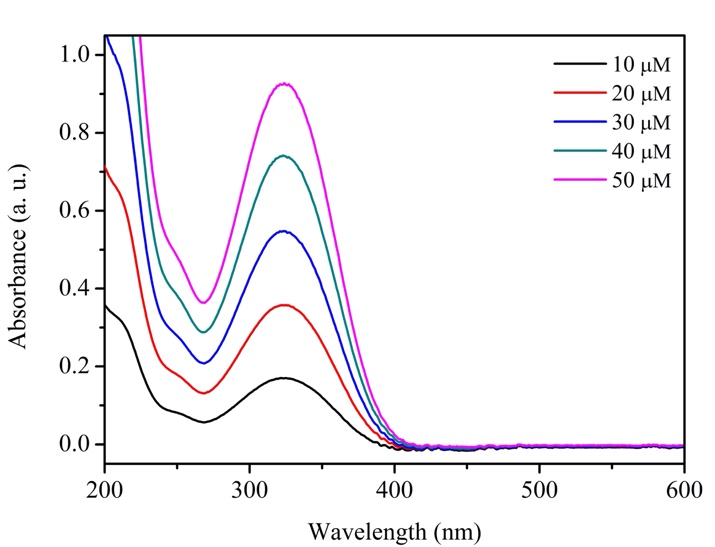


**Figure S5** UV-vis absorption spectra of DTNB at various concentrations (10 − 50 μM).


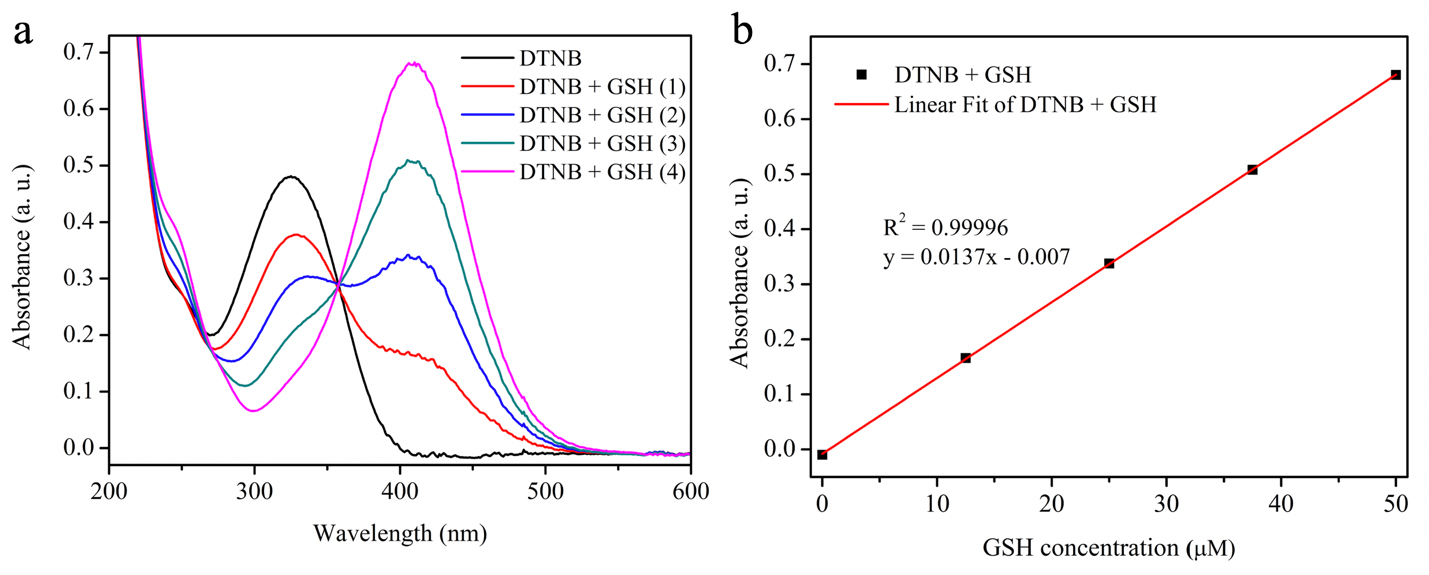


**Figure S6** (a) UV-vis absorption spectra of DTNB (25 μM) with various concentrations of GSH (12.5, 25, 37.5, and 50 μM). (b) Standard curve determined from (a) at 412 nm.


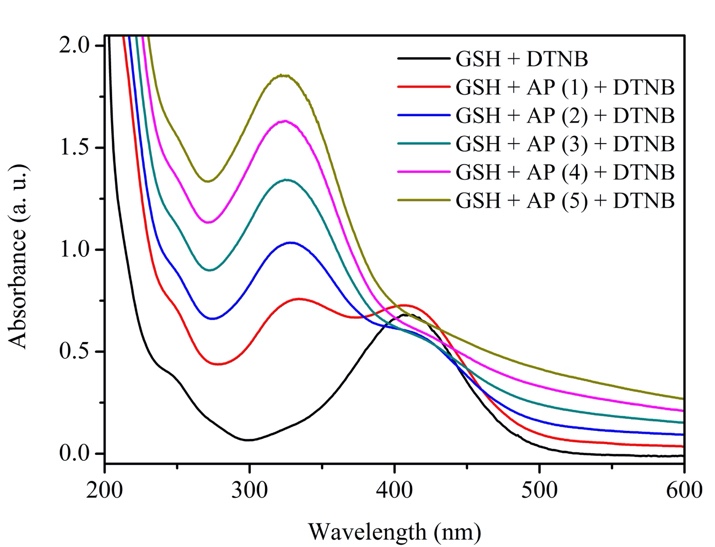


**Figure S7** Detection of GSH (50 μM) depletion by various concentrations of AP dispersions (5, 10, 15, 20 and 25 μg mL^−1^) after 12 h of reaction. [DTNB] = 25 μM.


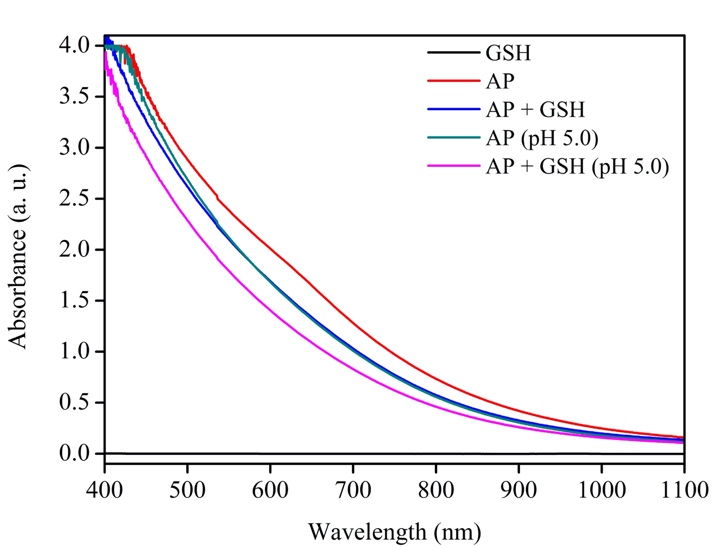


**Figure S8** The degradation of AP in GSH, acid (pH 5.0) and acidic GSH (pH 5.0) for 12 h. [GSH] = 1 mM, [AP] = 100 μg mL^−1^.


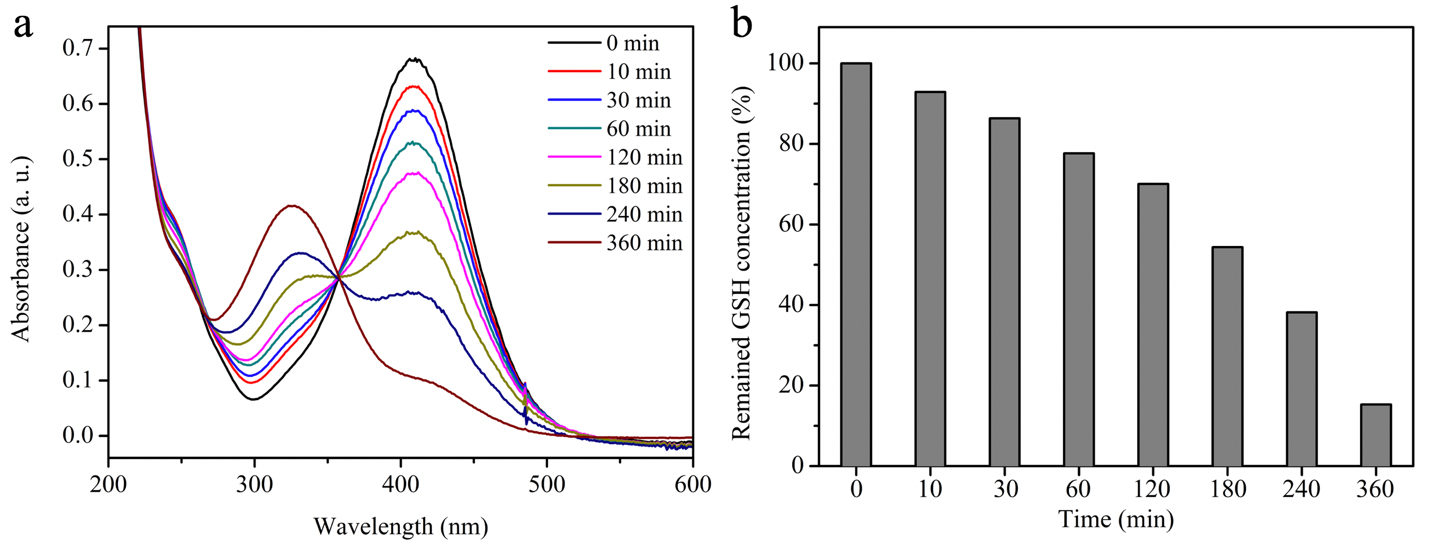


**Figure S9** Detection of GSH (50 μM) depletion by aqueous CuCl_2_ (50 μM) solution for 10, 30, 60, 120, 180, 240 and 360 min, respectively. [DTNB] = 25 μM.


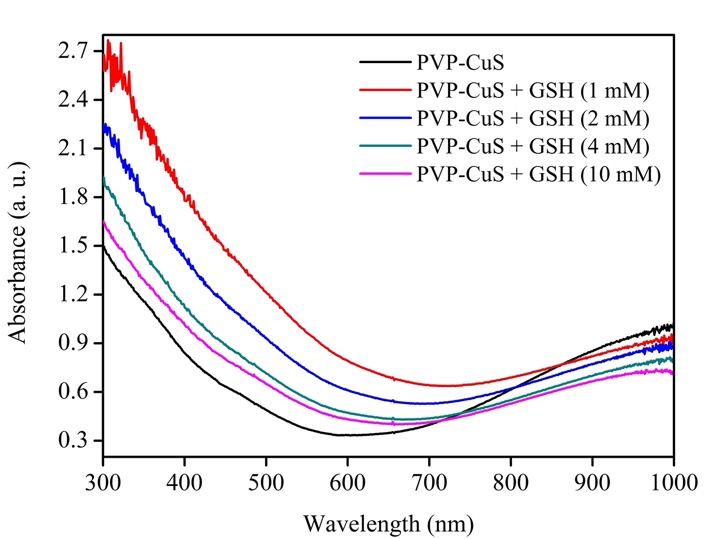


**Figure S10** UV-vis absorption spectra of PVP-CuS (25 μg mL^−1^) after incubation with various concentrations of GSH (0, 1, 2, 4 and 10 mM) for 6 h.


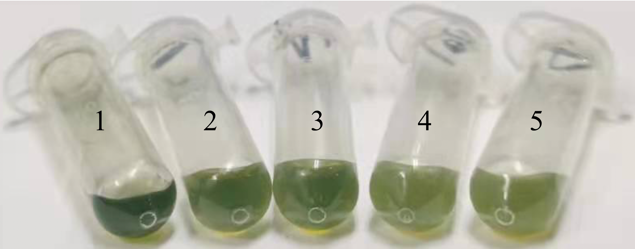


**Figure S11** Digital photos of PVP-CuS/GSH mixtures (separated by centrifugation and re-dispersed in 400 μL of DI H_2_O) after 6 h of reaction. [PVP-CuS] = 25 μg mL^−1^, [GSH] = (1) 0 mM, (2) 1 mM, (3) 2 mM, (4) 4 mM and (5) 10 mM.


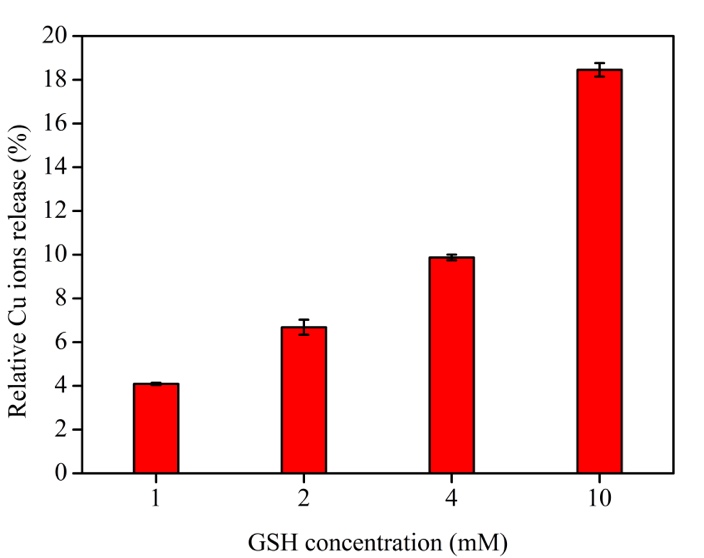


**Figure S12** Relative Cu ions release from PVP-CuS/GSH mixtures after incubation for 6 h. [PVP-CuS] = 25 μg mL^−1^, [GSH] = 1, 2, 4 and 10 mM.


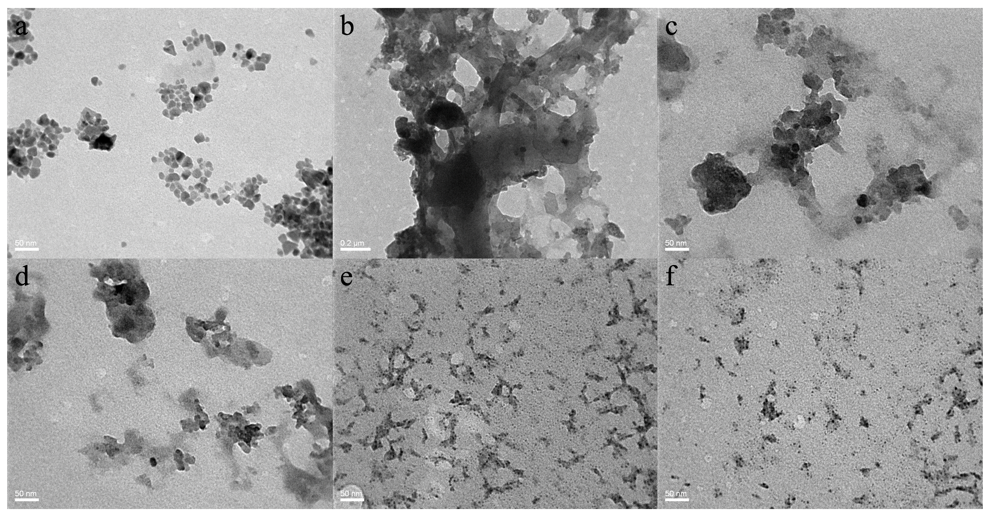


**Figure S13** TEM images of (a) PVP-CuS and (b-f) PVP-CuS/GSH mixtures after 24 h of reaction. [PVP-CuS] = 25 μg mL^−1^, [GSH] = 10 mM.


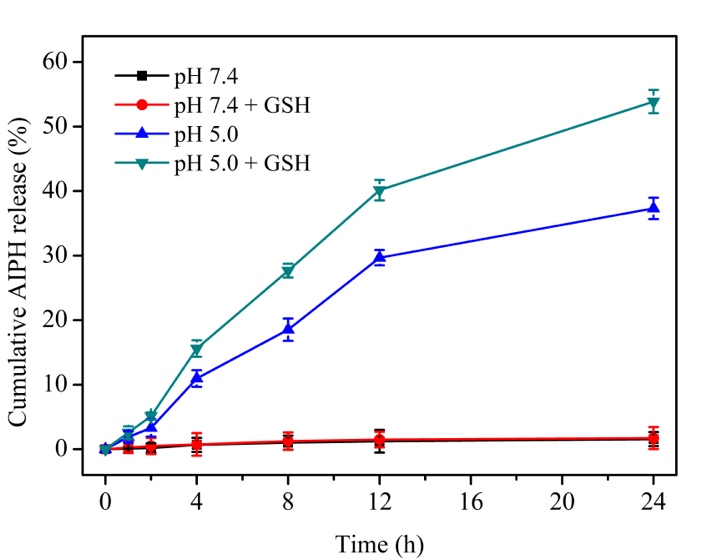


**Figure S14** Cumulative AIPH release profile of APCZ in PBS buffer of pH 7.4, pH 7.4 + GSH (10 mM), pH 5.0 and pH 5.0 + GSH (10 mM) for 24 h. Data shown as mean ± SD, n = 3 per treatment.


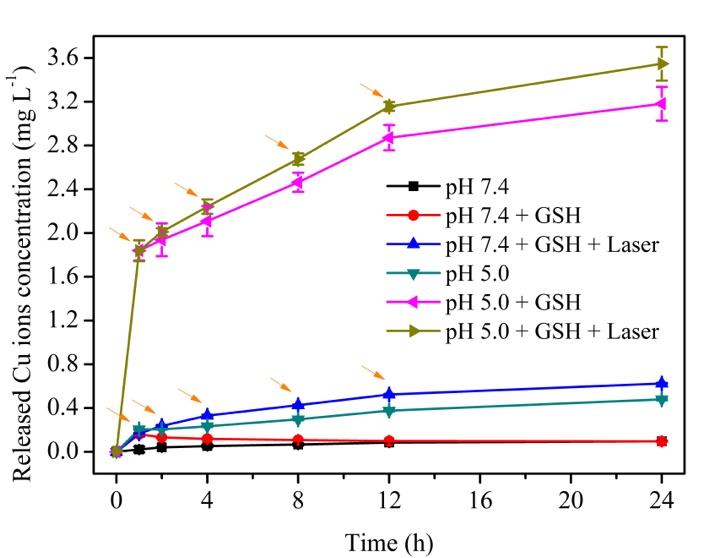


**Figure S15** Cumulative Cu ions release profile of APCZ in PBS buffer of pH 7.4, pH 7.4 + GSH (10 mM), pH 7.4 + GSH (10 mM) + Laser, pH 5.0, pH 5.0 + GSH (10 mM) and pH 5.0 + GSH (10 mM) + Laser. The orange arrows represented laser (1064 nm, 1.0 W cm^−2^) treatment, each time point was radiated for 10 min. Data shown as mean ± SD, n = 3 per treatment.


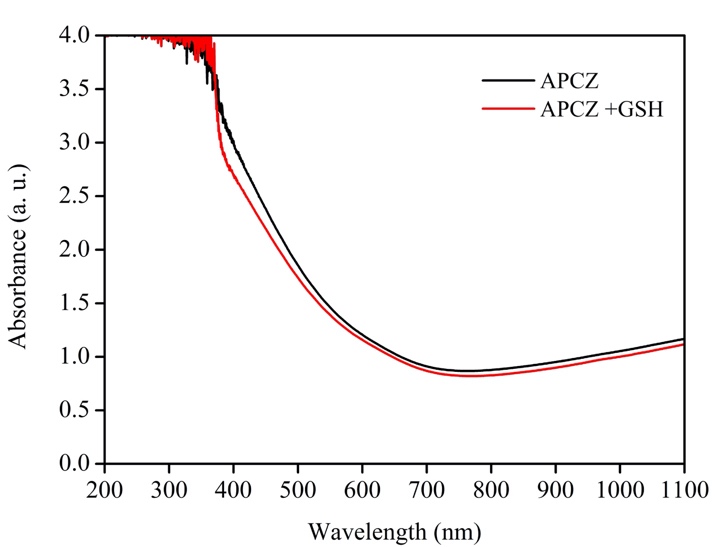


**Figure S16** UV-vis-NIR absorption spectra of APCZ and APCZ/GSH mixture after incubation in aqueous ABTS solution (44 °C) for 6 h. [APCZ] = 400 μg mL^−1^, [GSH] = 0.5 mM, [ABTS] = 20 μg mL^−1^.


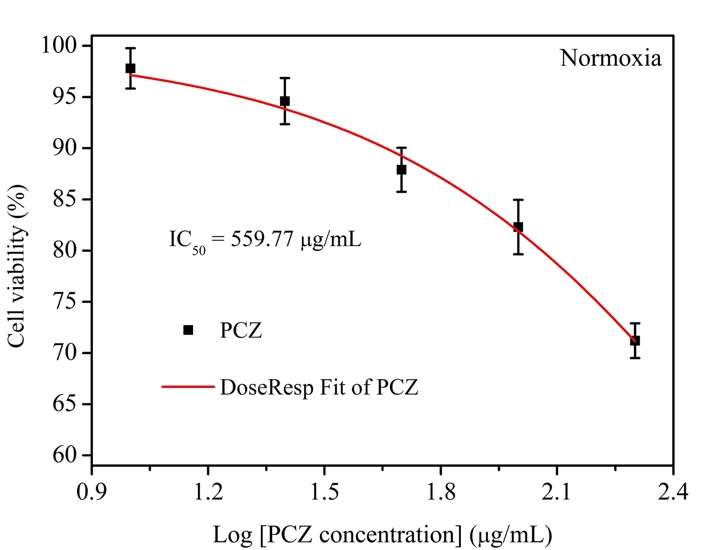


**Figure S17** IC_50_ of PCZ group in normoxic condition calculated from MTT results by GraphPad Prism 8 software.


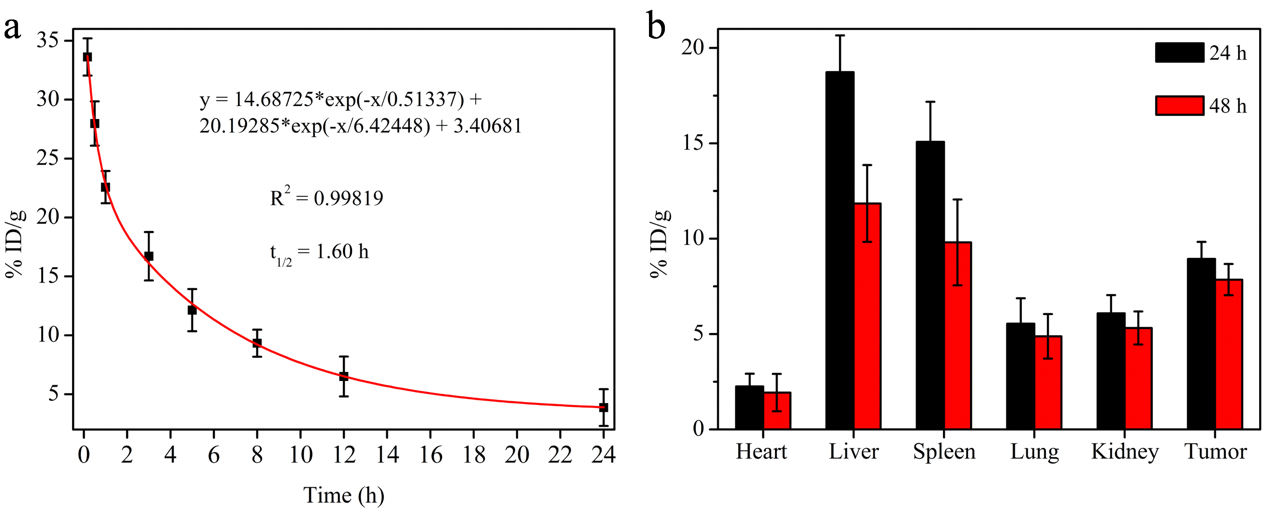


**Figure S18** (a) The blood clearance kinetics of APCZ after intravenously administration. (b) Biodistribution analysis of APCZ in 4T1 tumor bearing mice after the tail vein injection for 24 h and 48 h. Data shown as mean ± SD, n = 3 per treatment.


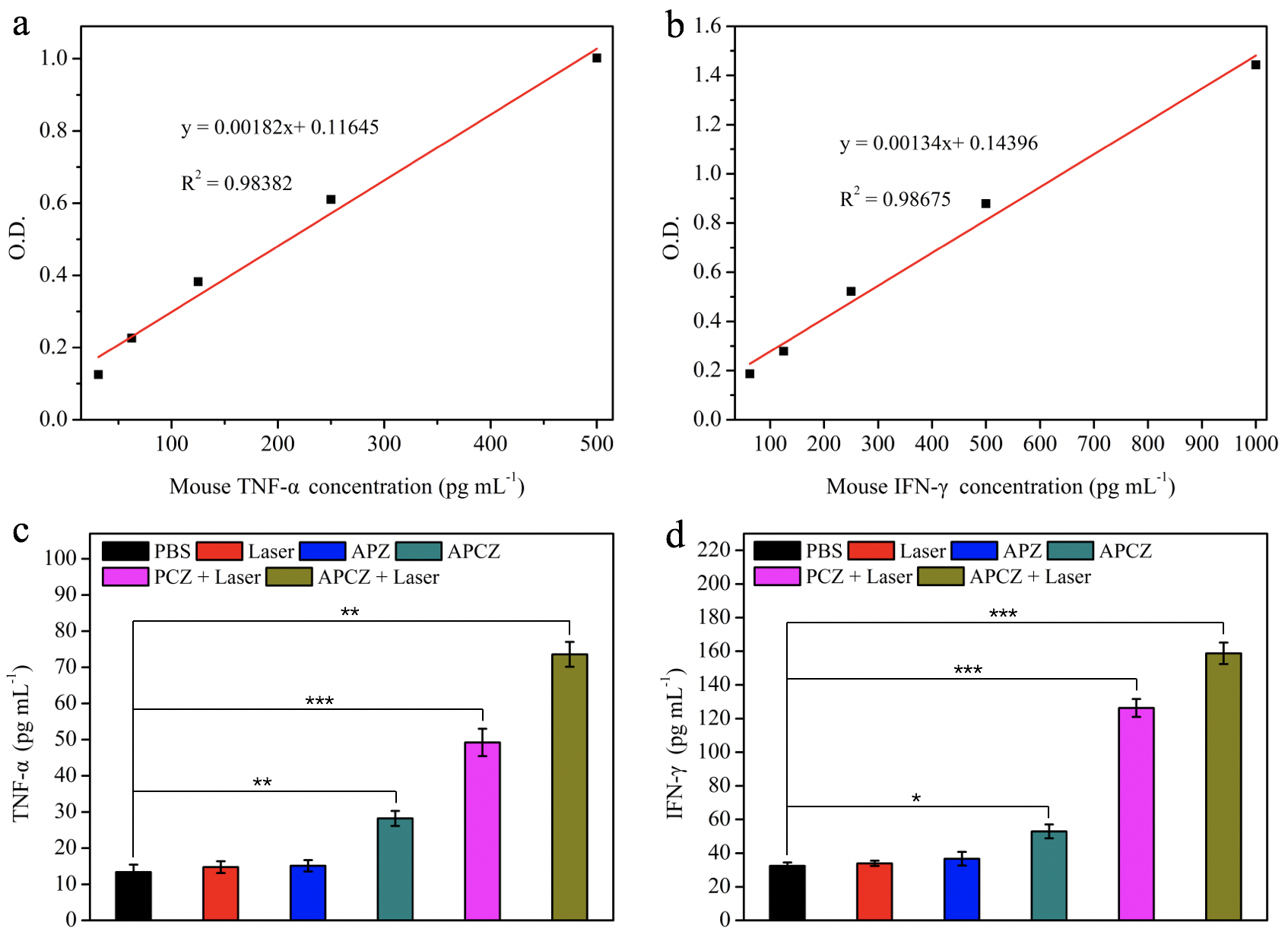


**Figure S19** Standard curves of (a) mouse TNF-α and (b) mouse IFN-γ. O.D. means optical density (absorbance at 450 nm). (c) TNF-α and (d) IFN-γ levels in sera isolated from different groups after 7-day treatments. Data shown as mean ± SD, n = 3 per treatment. Statistical significance was set at *p < 0.05, **p < 0.01, ***p < 0.001.


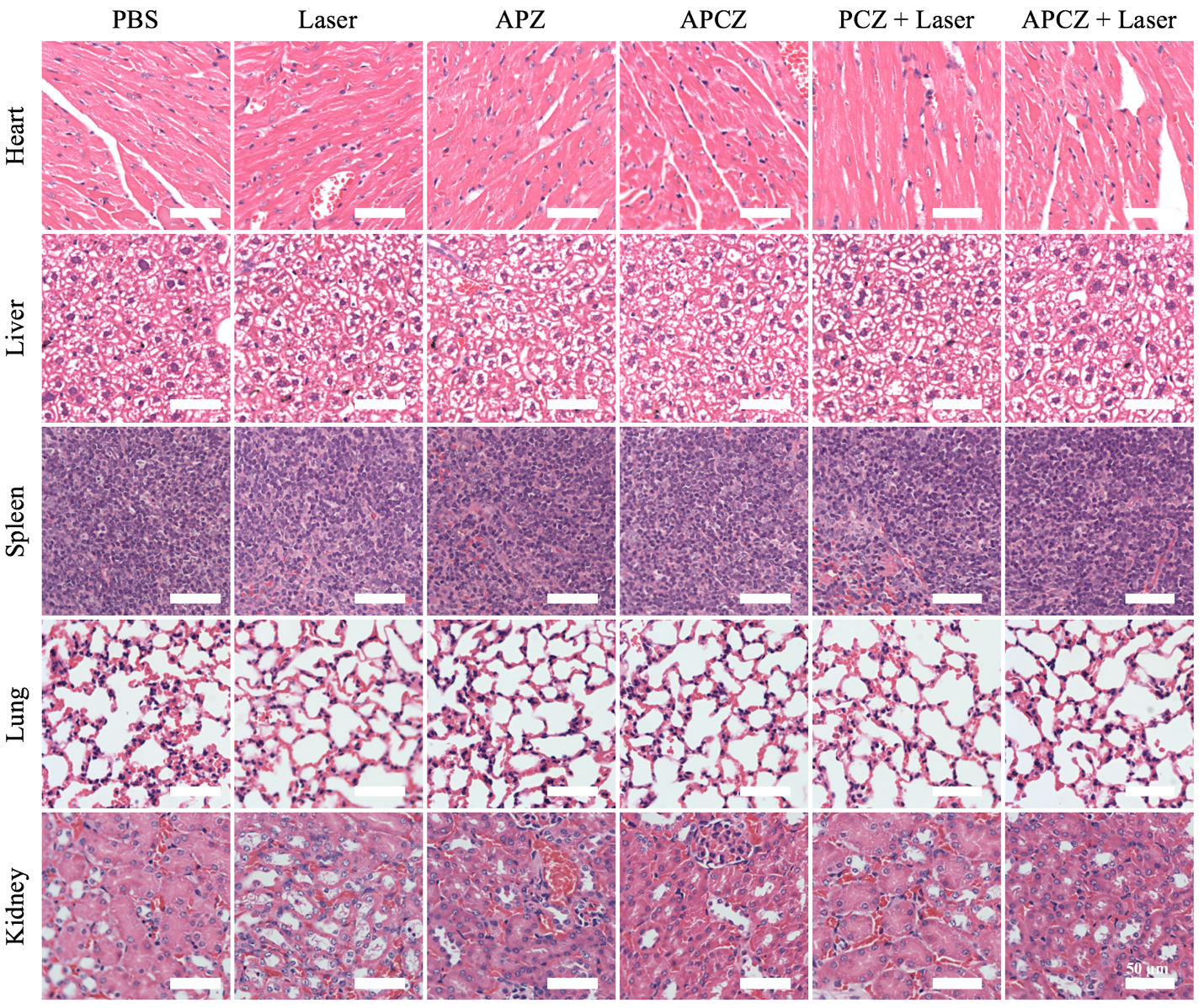


**Figure S20** H&E staining images of major organs after different treatments. Scale bars = 50 μm.

**Table S1** IC_50_ of different groups calculated from MTT results by GraphPad Prism 8 software.

| **Group** | **IC_50_ in normoxia** | **IC_50_ in hypoxia** |
| --- | --- | --- |
| PCZ | 559.77 μg mL^−1^ | 560.12 μg mL^−1^ |
| PCZ + Laser | 59.02 μg mL^−1^ | 61.32 μg mL^−1^ |
| APCZ + Laser | 49.09 μg mL^−1^ | 48.50 μg mL^−1^ |
